# TREM2 as a central hub of neuroimmune-metabolic crosstalk in central nervous system disorders: from microglial biology to therapeutic targeting

**DOI:** 10.3389/fimmu.2026.1912989

**Published:** 2026-08-20

**Authors:** Yulu Wu, Lian Duan, Luyu Zhang, Shuting Wang, Hongmu Yan, Mingyuan He, Tao Wang, Lili Li, Yuan Gao

**Affiliations:** 1 Department of Forensic Medicine, School of Basic Medicine, Soochow University, Suzhou, Jiangsu, China; 2 Department of Child and Adolescent Healthcare, Children’s Hospital of Soochow University, Suzhou, Jiangsu, China

**Keywords:** central nervous system diseases, microglia, neuroinflammation, therapeutic targets, TREM2

## Abstract

Triggering receptor expressed on myeloid cells 2 (TREM2) is a microglia-enriched immunoreceptor that functions as a central regulator of microglial adaptation by integrating immune surveillance, lipid sensing, metabolic reprogramming, and phagocytic responses in the central nervous system (CNS). Through association with the adaptor protein DAP12, TREM2 activates interconnected signaling networks involving the SYK pathway, PLCγ2-mediated Ca^2+^ signaling, PI3K-AKT-mTOR signaling, NF-κB activation and inflammatory regulatory pathways, thereby shaping microglial survival, migration, clearance capacity, and interactions with surrounding neural and immune cells. Increasing evidence from genetic studies, single-cell transcriptomics, spatial analyses, and human-derived microglial models indicates that TREM2 dysfunction contributes to diverse CNS disorders; however, its biological consequences are highly dependent on disease stage, pathological substrate, cellular context, and microenvironmental cues. In Alzheimer’s disease, TREM2 regulates amyloid-β-associated microglial responses, lipid metabolism, and synaptic remodeling, while its effects on tau-driven neurodegeneration remain controversial. In Parkinson’s disease, stroke, epilepsy, and amyotrophic lateral sclerosis, TREM2 influences α-Syn clearance, inflammatory resolution, tissue repair, and microglial state transitions, but may exert beneficial or maladaptive effects depending on temporal dynamics and disease-specific stressors. Emerging clinical evidence, including TREM2 variants, soluble TREM2 (sTREM2) biomarkers, and human multi-omics studies, highlights both the translational potential and complexity of targeting this pathway. Herein, this review summarizes the molecular mechanisms, physiological functions, and disease-specific roles of TREM2 in CNS disorders, critically discusses unresolved controversies and species-specific challenges, and evaluates emerging therapeutic strategies toward biomarker-guided and stage-specific modulation of TREM2 signaling. Understanding how to restore appropriate microglial adaptability rather than simply enhance or suppress TREM2 activity may provide a foundation for precision therapies in CNS disorders.

## 1 Introduction

Central nervous system (CNS) diseases represent a heterogeneous group of neurological conditions characterized by progressive alterations in neuronal function, immune regulation, and tissue homeostasis (1). These disorders encompass cerebrovascular diseases (2), neurodegenerative diseases (3), neuroinflammatory disorders (3), demyelinating diseases (4), movement disorders, and inherited neurological diseases, thereby imposing a substantial global health burden. Although their initiating triggers differ considerably, many CNS diseases converge on shared pathological processes, including chronic neuroinflammation, oxidative stress, metabolic dysfunction, impaired proteostasis, and defective clearance of cellular debris (3, 5). Current therapeutic strategies remain predominantly symptomatic, highlighting the urgent need to identify molecular regulators that integrate immune responses, metabolic adaptation, and tissue repair.

Microglia, the resident macrophages of the CNS, have emerged as central regulators of both physiological neural maintenance and disease-associated remodeling. Beyond their traditional role in immune surveillance, microglia actively regulate synaptic refinement, lipid metabolism, neuronal support, and injury-induced repair through dynamic changes in transcriptional, metabolic, and functional states (5–7). Under homeostatic conditions, microglia continuously monitor the neural environment and eliminate apoptotic cells and transient synaptic structures to maintain circuit integrity (8). Following pathological insults, however, microglia undergo extensive state transitions characterized by altered phagocytic capacity, inflammatory signaling, and metabolic reprogramming. Recent single-cell transcriptomic studies have revealed that disease-associated microglial states represent a spectrum of adaptive and maladaptive responses rather than discrete activation phenotypes. Nevertheless, the molecular determinants that govern whether microglial activation promotes tissue protection or contributes to chronic neurodegeneration remain incompletely understood.

Triggering receptor expressed on myeloid cells 2 (TREM2) has emerged as one of the most important regulators controlling microglial functional adaptation. As a lipid-sensing immune receptor predominantly expressed by microglia (9), TREM2 couples extracellular environmental signals to intracellular signaling pathways through its adaptor protein DNAX-activating protein 12 (DAP12, also known as TYROBP) (7). Activation of TREM2 regulates multiple aspects of microglial biology, including survival, proliferation, migration, phagocytosis, lipid processing, lysosomal function, and inflammatory responses (10). Notably, accumulating genetic, transcriptomic, and experimental evidence has positioned TREM2 as a critical determinant of disease-associated microglial programs across diverse CNS disorders (11). Rare coding variants in TREM2, particularly R47H, significantly increase the risk of Alzheimer’s disease (AD), highlighting its importance in human neurodegeneration (12). In AD, TREM2 regulates microglial responses to amyloid-β (Aβ), lipid accumulation, and synaptic remodeling; in Parkinson’s disease (PD), it influences α-Syn clearance, autophagy-lysosomal function, and inflammatory adaptation; in stroke, it contributes to debris removal and post-ischemic immune remodeling (13–15); whereas in amyotrophic lateral sclerosis (ALS), it regulates microglial state transitions in response to chronic motor neuron injury (16, 17).

Despite these advances, the biological and therapeutic interpretation of TREM2 remains highly complex. Increasing evidence indicates that TREM2 does not function as an intrinsically neuroprotective or pathogenic molecule. Instead, its consequences are determined by multiple interacting variables, including disease stage, pathological substrate, cellular context, signal intensity, metabolic status of microglia, and species-specific differences between experimental models and human disease. For example, TREM2 activation facilitates plaque containment and lipid adaptation in amyloid-driven AD models, yet persistent TREM2-associated microglial activation may contribute to maladaptive remodeling under chronic tau pathology or prolonged inflammatory stress. Similarly, reduced inflammatory cytokine production following TREM2 deficiency does not necessarily indicate therapeutic benefit, as impaired phagocytosis, metabolic failure, and defective tissue surveillance may coexist with attenuated inflammatory signatures. These observations highlight a fundamental unresolved question: how can TREM2 signaling be therapeutically modulated to restore beneficial microglial functions while avoiding maladaptive activation?

Addressing this question requires integration of evidence from experimental models and human studies. Although animal models have provided important mechanistic insights into TREM2 signaling, they incompletely reproduce human microglial biology, aging-associated changes, genetic diversity, and disease heterogeneity. Recent advances in human single-cell RNA sequencing, spatial transcriptomics, human induced pluripotent stem cell (iPSC)-derived microglia, and longitudinal biomarker studies have begun to redefine TREM2 biology in human neurological disorders. These approaches provide critical opportunities to identify disease-relevant microglial states, clarify the relationship between membrane-bound TREM2 and sTREM2, and establish clinically actionable biomarkers for patient stratification.

In this review, we summarize current advances in TREM2 biology, including its molecular structure, expression patterns, physiological functions, and signaling networks. We then critically discuss the disease-specific roles of TREM2 in epilepsy, stroke, AD, PD, and ALS, with particular emphasis on the mechanisms underlying its context-dependent and stage-specific effects. Furthermore, we evaluate emerging therapeutic strategies targeting TREM2, including receptor agonism, indirect pathway modulation, gene-based approaches, and microglial replacement strategies, while highlighting the challenges associated with clinical translation. By integrating mechanistic discoveries with emerging human evidence, this review aims to provide a conceptual framework for understanding TREM2 as a dynamic regulator of microglial adaptability and to define future directions for precision therapeutic intervention in CNS disorders.

## 2 Biological characteristics of TREM2

### 2.1 Molecular structure and function of TREM2

TREM2 is a member of the immunoglobulin superfamily and a key innate immune receptor predominantly expressed by microglia in the CNS (18). TREM2 is encoded by a gene located on human chromosome 6p21.1 (mouse chromosome 17C3) and comprises five exons that generate a cDNA of approximately 693 base pairs, encoding a 230-amino-acid glycoprotein with a predicted molecular weight of 25–30 kDa (19). Structurally, TREM2 consists of four distinct domains: an N-terminal signal peptide (SP; amino acids 1-18), an extracellular domain (ECD; amino acids 19-173), a transmembrane domain (TM; amino acids 174-195), and a short intracellular domain (ICD; amino acids 196-230). The extracellular region, encoded primarily by exon 2, contains a characteristic V-set immunoglobulin (IgV) domain and a stalk region (20). Within the IgV domain, three complementarity-determining regions (CDR1-3) form the principal ligand-binding interface, enabling TREM2 to recognize a broad spectrum of endogenous ligands, including phospholipids, apolipoproteins, lipoprotein particles, and damage-associated molecules (21). Notably, the ETD contains three N-linked glycosylation sites. Glycosylation substantially influences receptor maturation, membrane trafficking, ligand recognition, and protein stability, increasing the apparent molecular mass of TREM2 from approximately 26 kDa to 36–60 kDa (22).

Unlike many immune receptors, TREM2 possesses only a short cytoplasmic tail and lacks intrinsic signaling motifs. Consequently, its signaling capacity depends on association with the transmembrane adaptor protein DAP12 (23). Upon ligand engagement, TREM2 forms a functional receptor complex with DAP12, leading to phosphorylation of the immunoreceptor tyrosine-based activation motif (ITAM) within DAP12 and subsequent recruitment of spleen tyrosine kinase (SYK) (24). This event initiates a cascade of downstream signaling pathways, including PI3K-Akt, PLCγ2, ERK/MAPK, and mTOR signaling, which collectively regulate microglial survival, proliferation, migration, phagocytosis, metabolic adaptation, and inflammatory responses (25). Accumulating evidence indicates that TREM2 functions as both an immune receptor and a lipid-sensing immunometabolic regulator. In the healthy CNS, TREM2 facilitates the uptake and processing of lipids, promotes cholesterol efflux, supports myelin debris clearance, and enhances the removal of neurotoxic protein aggregates such as Aβ. These functions support microglial homeostasis and tissue integrity (26–28). Under pathological conditions, TREM2 signaling orchestrates microglial phenotypic transitions, including the emergence of disease-associated microglia (DAM) (14), thereby reshaping inflammatory and metabolic programs in response to tissue injury and neurodegeneration. Given its central role in integrating lipid sensing, immune signaling, and microglial functional remodeling, TREM2 has emerged as a pivotal molecular hub linking neuroinflammation, metabolic homeostasis, and neurodegenerative pathology (29). Consequently, TREM2 is increasingly recognized as a promising therapeutic target for a broad spectrum of CNS disorders.

### 2.2 TREM2 variants: molecular consequences and disease relevance

Genetic variation is a major determinant of TREM2 function and contributes substantially to inter-individual susceptibility to CNS disorders. TREM2 polymorphisms influence receptor expression, post-translational modification, ligand recognition, membrane localization, and downstream signaling, thereby shaping microglial responses and neuroimmune homeostasis.

At the transcript level, three major TREM2 isoforms have been identified in the human brain (30, 31). The longest transcript (ENST00000373113) contains all five exons and encodes the full-length receptor with an intact transmembrane domain, representing the predominant form used in functional studies. A second isoform (ENST00000373122) lacks exon 5 but retains membrane-anchoring capacity and signaling competence. In contrast, the shortest transcript (ENST00000338469) lacks exon 4 and the transmembrane domain, giving rise to a soluble protein isoform known as sTREM2 (32). In addition to this splice-derived soluble form, sTREM2 can also be generated through proteolytic shedding of membrane-bound TREM2 by ADAM family metalloproteinases, primarily ADAM10 and ADAM17 (33). Thus, alternative splicing and ectodomain shedding represent two distinct but complementary mechanisms for generating soluble forms of TREM2. Increasing evidence suggests that sTREM2 is not merely a degradation product but an active signaling molecule capable of modulating microglial survival, activation, phagocytosis, and inflammatory responses. Elevated levels of sTREM2 have been detected in cerebrospinal fluid (CSF) from patients with AD, PD, MS, and progranulin (GRN)-associated frontotemporal dementia (FTD) (32), where they are closely associated with disease stage, neurodegenerative biomarkers, and genetic risk (34).

At the protein level, numerous coding variants have been identified that alter TREM2 structure and function. These variants influence key biological processes including glycosylation, proteolytic processing, ligand binding, and receptor signaling. For example, mutations affecting residue 20 impair glycosylation (35), whereas substitutions at residues 36 and 60 reduce ADAM10-mediated ectodomain shedding and alter sTREM2 generation. Variants affecting residue 48 impair binding to lipoprotein-associated ligands, including ApoE, clusterin (CLU), and low-density lipoproteins (LDL), thereby compromising lipid sensing and downstream signaling. Although most disease-associated variants occur within coding regions, regulatory variants have also been identified in the 3′ untranslated region and promoter-proximal sequences, suggesting additional mechanisms of transcriptional regulation.

Among all identified variants, R47H (rs75932628) is the most extensively studied and represents one of the strongest genetic risk factors for late-onset AD (36, 37). Located within the ligand-binding IgV domain, R47H markedly reduces ligand recognition, impairs microglial phagocytosis, and alters CSF tau profiles. Other disease-associated variants include R62H, D87N, T96K, R136Q, and L211P (38, 39). Functionally, most variants exhibit partial loss-of-function phenotypes, whereas T96K appears to enhance ligand affinity and downstream signaling activity. Overall, these findings highlight considerable functional heterogeneity among TREM2 variants (**Table 1**).

**Table 1:** TREM2 genetic variants and their molecular consequences in CNS disorders.

Genetic variant	Amino acid change	Functional consequence	Associated CNS disorders	References
40G→T	E14X	LOF	NHD	(43)
97C→A	Q33X	LOF	NHD,FTD	(43, 44)
132G→A	W44X	LOF	NHD	(44)
233G→A	W78X	LOF	NHD	(44, 45)
588G→A	K186N	Partial LOF	NHD	(46)
401A→G	D134G	Partial LOF	NHD	(47)
377T→G	V126G	Partial LOF	NHD	(47)
113A→G	Y38C	Partial LOF	NHD,FTD	(48)
140C→T	R47H	Partial LOF	AD,PD,ALS,FTD	(45, 49, 50)
197C→T	T66M	Partial LOF	NHD,FTD	(48)
257A→T	D86V	Partial LOF	FTD	(44, 49)
286C→A	T96K	GOF	AD,FTD	(48)
598G→A	W198X	LOF	FTD	(51)
185C→T	R62H	Partial LOF	AD	(48, 49)
259C→T	D87N	Partial LOF	AD	(52)
469C→T	H157Y	Partial LOF	AD	(50, 51)
632C→T	L211P	Partial LOF	AD	(48)

Specifically, the pathogenic consequences of TREM2 variants are highly disease-dependent. In Nasu-Hakola disease (NHD) (40, 41), loss-of-function mutations frequently cause severe protein misfolding, defective trafficking, or complete absence of functional TREM2 (21), resulting in early-onset dementia accompanied by bone cysts. In contrast, AD-associated variants such as R47H generally preserve membrane expression but exhibit impaired ligand binding and signaling (42), leading to partial functional deficits that contribute to slowly progressive neurodegeneration. These observations indicate that distinct TREM2 variants generate a spectrum of functional impairment rather than a uniform loss-of-function state.

Overall, TREM2 genetic variation serves as a critical molecular link between inherited susceptibility and dysregulated neuroimmune-metabolic responses. By influencing receptor processing, ligand sensing, and microglial activation states, TREM2 variants reshape disease trajectories across diverse CNS disorders and provide a strong rationale for the development of genotype-informed therapeutic strategies.

### 2.3 Endogenous and exogenous ligands of TREM2: linking lipid sensing to immunometabolic signaling

The biological functions of TREM2 are initiated by its ability to recognize a remarkably diverse repertoire of endogenous and exogenous ligands. Through ligand sensing, TREM2 serves as a molecular interface that links environmental cues to intracellular immunometabolic responses, thereby regulating microglial activation, phagocytosis, survival, and tissue homeostasis. Within this diverse ligand repertoire, endogenous lipid-associated molecules constitute the most physiologically relevant class. TREM2 was initially characterized as a lipid-sensing receptor capable of recognizing anionic and zwitterionic lipids exposed during cellular stress, apoptosis, or tissue injury (12, 53, 54). One of the best-characterized ligands is phosphatidylserine (PS), which becomes exposed on the outer leaflet of apoptotic cells and damaged membranes. Recognition of PS enables microglia to detect and clear apoptotic cells, myelin debris, and degenerating neuronal structures, thereby contributing to tissue remodeling and homeostatic maintenance. In addition to phospholipids, TREM2 interacts with a broad spectrum of lipoprotein-associated ligands, including low-density lipoprotein (LDL), apolipoprotein A-I (ApoA-I), and clusterin (CLU) (55).

Among these ligands, apolipoprotein E (ApoE) (50) has emerged as a central physiological and pathological ligand of TREM2. ApoE directly binds both membrane-bound TREM2 and sTREM2 in a residue-dependent manner and plays a crucial role in regulating microglial responses to lipid accumulation and protein aggregation (56). Through the ApoE-TREM2 axis, microglia acquire enhanced phagocytic capacity, increased lipid-processing ability, and a disease-associated transcriptional program that promotes the clearance of amyloid plaques, apoptotic debris, and damaged myelin. Consequently, ApoE-TREM2 signaling is increasingly recognized as a major molecular bridge linking neurodegeneration, lipid metabolism, and innate immune responses.

Beyond lipid ligands, TREM2 can also recognize a variety of endogenous danger-associated molecules. These include nucleic acids, heat shock protein 60 (HSP60) (57), and cellular debris released from stressed or dying cells. Such molecules function as indicators of tissue damage and contribute to the activation of microglial surveillance and repair programs. In neurodegenerative conditions, disease-associated ligands-including Aβ aggregates, apoptotic lipid particles, myelin-derived lipids (25), and extracellular DNA released from necrotic cells-further stimulate TREM2-dependent signaling pathways, promoting adaptive microglial responses to pathological stress (58–60). Although these endogenous ligands appear to predominate in the CNS, TREM2 can also recognize several exogenous ligands, suggesting a broader role in innate immune surveillance. Early studies demonstrated that TREM2 binds to negatively charged bacterial components derived from both Gram-positive and Gram-negative bacteria, including LPS (61) and lipoteichoic acid (LTA). Interestingly, TREM2 exhibits selective recognition of microbial products, binding efficiently to certain bacterial species such as Escherichia coli but displaying limited affinity for others, including Salmonella. These observations suggest that TREM2 may contribute to innate immune surveillance while maintaining functional specificity toward distinct microbial signals.

Ligand engagement triggers the formation of a signaling complex involving TREM2 and the adaptor proteins DAP12 and DAP10 (53). Phosphorylation of the ITAM within DAP12 recruits SYK kinase and subsequently activates multiple downstream pathways (62), including PI3K-Akt (63), PLCγ, ERK/MAPK, mTOR, and calcium-dependent signaling cascades. These pathways collectively regulate microglial survival, proliferation, migration, phagocytosis, inflammatory responses, and metabolic adaptation. Interestingly, accumulating evidence suggests that distinct ligands can induce quantitatively and qualitatively different signaling outputs. Lipid-rich ligands generally favor phagocytic and metabolic programs, whereas certain inflammatory stimuli preferentially modulate cytokine production and immune activation. Conversely, weak or transient stimulation may recruit inhibitory molecules such as SHIP1, thereby dampening excessive inflammatory signaling and maintaining immune homeostasis.

Converging evidence supports the notion that TREM2 functions as a versatile environmental sensor that integrates signals derived from lipids, lipoproteins, damaged cells, protein aggregates, and microbial products. Through its interaction with DAP12-dependent signaling pathways, ligand recognition initiates a coordinated immunometabolic response that governs microglial behavior in both physiological and pathological conditions. This ligand-driven signaling framework provides the mechanistic foundation for the role of TREM2 in neuroimmune communication, lipid metabolic reprogramming, and CNS disease progression.

### 2.4 Multi-layered regulation of TREM2 activity

TREM2 activity is tightly controlled through multiple regulatory layers, including post-translational modification, proteolytic processing, transcriptional regulation, and inflammatory signaling. These mechanisms collectively determine receptor abundance, ligand responsiveness, downstream signaling intensity, and ultimately microglial functional states under both physiological and pathological conditions.

At the post-translational level, the extracellular immunoglobulin-like domain of TREM2 plays a pivotal role in receptor maturation and ligand recognition. This region contains multiple N-glycosylation sites that are essential for proper protein folding, structural stability, and ligand binding (21). Glycosylation-dependent conformational maturation facilitates the formation of intramolecular disulfide bonds, including Cys36-Cys110 and Cys51-Cys60, thereby stabilizing the ligand-binding interface and promoting the assembly of the TREM2-DAP12 signaling complex (23–25). Consequently, alterations in glycosylation status can profoundly influence receptor trafficking, ligand affinity, and downstream signal transduction. Once mature TREM2 reaches the cell surface, its abundance and persistence are further controlled by sequential proteolytic cleavage. Membrane-bound TREM2 undergoes ectodomain shedding primarily through the metalloproteinases ADAM10 and ADAM17, which cleave the receptor at the His157-Ser158 site and release sTREM2 into the extracellular environment. Following ectodomain shedding, the remaining membrane-associated C-terminal fragment is further processed by γ-secretase (64), generating an intracellular domain that is subsequently degraded. Increasing evidence suggests that this sequential cleavage process is not merely a degradation pathway but a dynamic regulatory mechanism controlling receptor availability and signaling duration. Moreover, sTREM2 itself has emerged as a biologically active molecule capable of modulating microglial survival, inflammatory responses, and phagocytic activity. Therefore, the ADAM10/17-γ-secretase axis represents a central checkpoint governing TREM2 function (3).

At the transcriptional level, TREM2 expression is regulated by a complex network of lineage-specific transcription factors and signaling pathways. PU.1, a master regulator of myeloid-cell identity, directly binds the TREM2 promoter and serves as a key determinant of basal TREM2 expression (65, 66). In contrast, activation of NF-κB signaling can suppress TREM2 expression indirectly through the induction of microRNA-34a (67). Additional regulators, including retinoid X receptor (RXR), NFAT, protein E, and receptor activator of nuclear factor-κB ligand (RANKL), have also been implicated in the modulation of TREM2 transcription in specific cellular contexts, although the underlying mechanisms remain incompletely understood. This transcriptional network does not operate in isolation, as genetic and compensatory relationships among TREM family members may also influence the overall balance of TREM2 signaling. Genetic studies have shown that variants in TREML2 and TREML4 can influence the expression of TREM2 and TREML1 within the brain (66). Likewise, AD-associated TREM1 variants are frequently accompanied by reduced TREM1 expression and compensatory upregulation of TREM2 in circulating monocytes (68). These observations suggest that functional interactions among TREM family members may shape innate immune responses and that the balance between TREM1 and TREM2 signaling could be more biologically relevant than the absolute expression level of either receptor alone (69).

Inflammatory and environmental signals provide an additional layer of regulation. Pro-inflammatory mediators-including TNF-α, IL-1β, interferon-γ, reactive oxygen species (ROS), TLR agonists, and LPS-generally suppress TREM2 expression and signaling activity (70). By contrast, anti-inflammatory factors such as IL-4 and vasoactive intestinal peptide promote TREM2 expression and support microglial homeostasis (71). Interestingly, discrepancies have been observed between *in vitro* and *in vivo* studies. Whereas acute inflammatory stimulation often reduces TREM2 expression in cultured cells, increased TREM2 levels are frequently detected in diseased brain tissues (72). This apparent paradox may reflect the combined effects of microglial recruitment, chronic inflammatory adaptation, cellular heterogeneity, and disease-associated phenotypic transitions.

**Figure 1:**
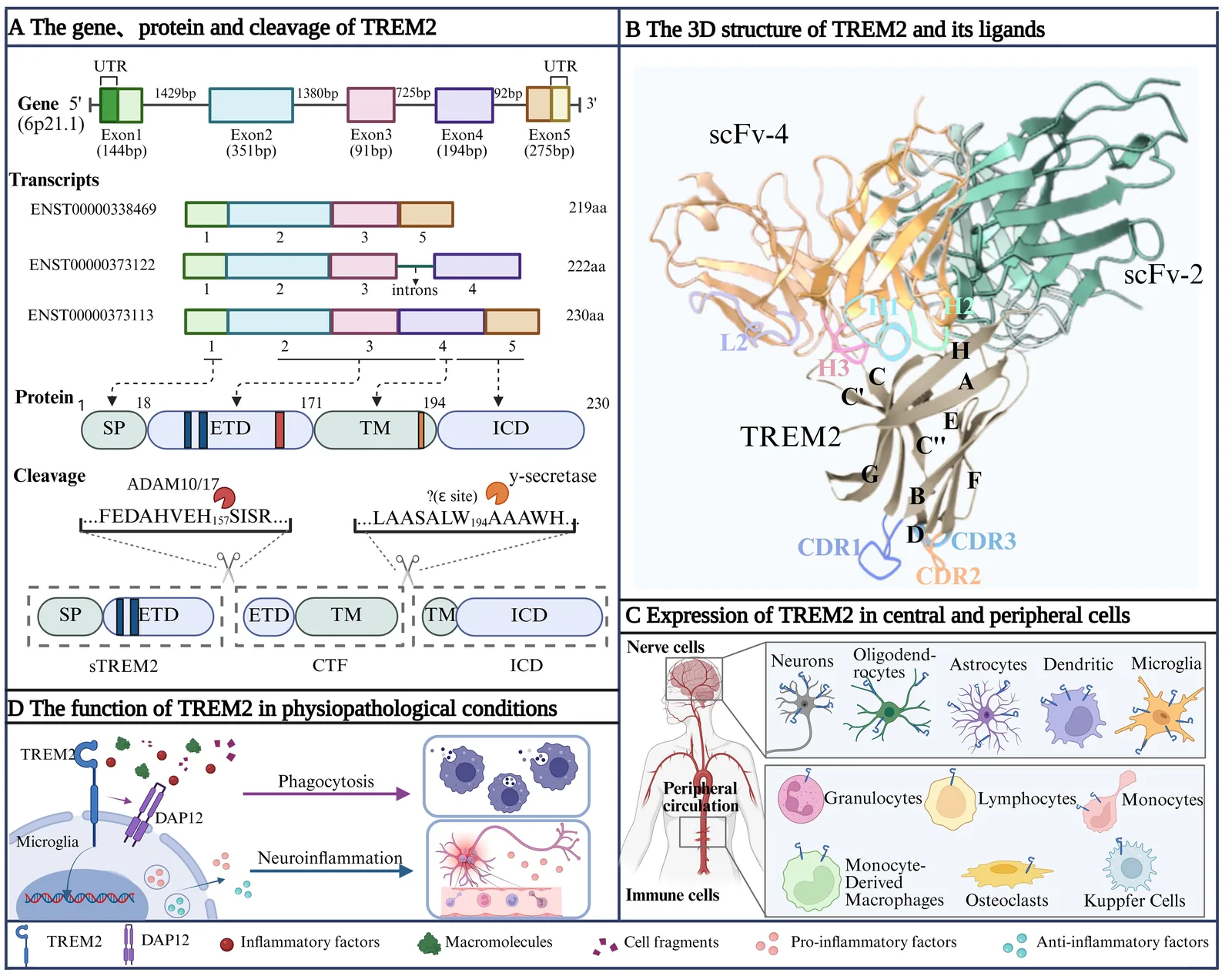
Structural characteristics, cellular distribution and physiological functions of TREM2 **(A)** Genomic organization and protein structure of TREM2. Human TREM2 is located on chromosome 6p21.1, whereas the murine Trem2 gene is located on chromosome 17C3. The gene contains five exons and encodes a 230-amino-acid type I transmembrane glycoprotein (~25–30 kDa), consisting of a signal peptide (SP, residues 1-18), an extracellular immunoglobulin-like domain (ECD, residues 19-173), a transmembrane domain (TM, residues 174-195), and a short intracellular domain (ICD, residues 196-230). **(B)** Crystal structure of TREM2 in complex with its binding antibodies (scFv-2 and scFv-4), illustrating the architecture of the extracellular ligand-binding domain and key structural determinants involved in receptor recognition. **(C)** Cellular distribution of TREM2 in the central and peripheral immune systems. Within the CNS, TREM2 is predominantly expressed by microglia, with lower expression reported in neurons, astrocytes, and oligodendrocytes under specific physiological or pathological conditions. In peripheral tissues, TREM2 is mainly detected in monocyte-derived macrophages, dendritic cells, osteoclasts, and other myeloid-lineage cells. Through these cellular populations, TREM2 contributes to intercellular communication and immune homeostasis. **(D)** Physiological functions of TREM2 in the neuroimmune microenvironment. Upon association with the adaptor protein DAP12, TREM2 regulates microglial activation, migration, survival, and phagocytic clearance of apoptotic cells, myelin debris, protein aggregates, and other extracellular substrates. In addition, TREM2 participates in the modulation of neuroinflammatory responses and maintenance of CNS homeostasis through coordinated interactions with neurons, astrocytes, and peripheral immune cells.

Overall, TREM2 activity is governed by a multilayered regulatory network spanning receptor maturation, ectodomain shedding, transcriptional control, and environmental signaling. These regulatory mechanisms ensure precise spatiotemporal control of TREM2-dependent immunometabolic responses and provide multiple therapeutic entry points for modulating microglial function in CNS disorders (**Figure 1**).

## 3 Pathophysiological functions and signaling networks of TREM2

### 3.1 TREM2 in phagocytosis and tissue surveillance

A fundamental physiological function of TREM2 is to regulate microglial phagocytic surveillance, a process essential for maintaining CNS homeostasis. Efficient recognition and removal of apoptotic cells, extracellular protein aggregates, damaged myelin, and cellular debris prevent secondary necrosis and limit the persistent activation of innate immune pathways. Consistent with this role, TREM2 is highly expressed in microglia and other tissue-resident myeloid populations characterized by active lipid sensing, phagocytosis, and metabolic adaptation.

Accumulating evidence demonstrates that TREM2 functions as a critical regulator of substrate recognition, engulfment efficiency, and phagocytic adaptation. Genetic deletion of TREM2 impairs the uptake of apoptotic neurons, lipid-rich debris, myelin fragments, and pathogenic protein aggregates, whereas enhanced TREM2 signaling increases microglial clearance capacity. In AD models, TREM2 expression positively correlates with microglial uptake and containment of Aβ42, whereas TREM2-deficient microglia exhibit impaired Aβ internalization and reduced plaque-associated clustering (42). However, increasing evidence indicates that TREM2 does not simply act as a universal enhancer of phagocytosis. Instead, TREM2 regulates the threshold, selectivity, and metabolic sustainability of microglial engulfment responses. For example, inflammatory stimulation by LPS can disrupt the relationship between TREM2 expression and Aβ phagocytosis, suggesting that TREM2-dependent clearance programs are highly dependent on the surrounding inflammatory and metabolic environment.

Recent studies have further revealed that TREM2-mediated phagocytosis is tightly coupled with metabolic reprogramming. For example, cyanidin-3-glucoside enhances TREM2 surface expression in Aβ-treated HMC3 microglial cells, promotes Aβ uptake, and facilitates microglial functional adaptation through PPARγ-dependent pathways (73, 74). These findings support the concept that TREM2-mediated phagocytosis is not an isolated engulfment process but rather a coordinated immune-metabolic program involving lipid handling, mitochondrial adaptation, and lysosomal function. Importantly, TREM2-dependent surveillance operates within a complex neuro-glial communication network rather than through an autonomous microglial pathway (6). Within the neuro-glial unit, microglia, astrocytes, neurons, and extracellular matrix components collectively regulate tissue remodeling. TREM2 signaling influences this network by coordinating microglial responses with astrocytic and neuronal signals. For example, microglia-derived inflammatory and trophic factors can reshape astrocytic responses, whereas astrocyte-derived extracellular nucleotides activate purinergic receptors on microglia, including P2Y12 and P2Y6, thereby modulating calcium-dependent phagocytic activity (75). These interactions highlight that TREM2 functions as an integrative sensor that coordinates multicellular responses during tissue surveillance.

A key concept emerging from recent studies is that TREM2-mediated phagocytic surveillance represents a biological continuum between physiological remodeling and pathological synaptic elimination. During development, microglia eliminate transient or weakened synaptic connections to refine neural circuits. TREM2 contributes to this process by regulating microglial recognition and engulfment programs that cooperate with complement-dependent tagging mechanisms, including C1q-associated synaptic labeling. Importantly, this process is tightly controlled by neuronal activity, lipid-derived signals, and inhibitory feedback mechanisms, ensuring selective removal of inappropriate synapses while preserving functional connections. Thus, developmental synaptic pruning reflects a regulated surveillance program rather than indiscriminate phagocytosis.

In neurodegenerative diseases, synaptic loss does not appear to involve the activation of an entirely new phagocytic pathway; rather, it represents a pathological deviation of an existing TREM2-dependent remodeling program. Recent human iPSC-derived microglial co-culture studies demonstrated that TREM2 contributes to the recognition and engulfment of synapses displaying apoptotic-like stress signals in AD (76). Consistent with human genetic and transcriptomic studies, TREM2-expressing microglial populations are enriched in disease-associated regions of AD brains, particularly around amyloid plaques. However, whether this response is beneficial or detrimental depends on the pathological environment. Under conditions characterized by controlled lipid metabolism and efficient lysosomal processing, TREM2 activation may facilitate removal of damaged synapses and restoration of tissue homeostasis. Conversely, in the presence of chronic lipid overload, complement overactivation, persistent inflammatory signaling, or impaired metabolic flexibility, the same phagocytic program may become maladaptive and contribute to excessive synaptic elimination.

This physiological-to-pathological transition illustrates an important principle of TREM2 biology: TREM2 does not intrinsically promote either neuroprotection or neurodegeneration; instead, it determines the activation threshold and functional outcome of microglial surveillance according to cellular state and environmental context. This concept may explain why TREM2 activation is beneficial in some conditions, such as developmental remodeling and early amyloid pathology, but may contribute to disease-associated synapse loss during chronic neurodegeneration.

Although TREM2 is essential for maintaining phagocytic competence, accumulating evidence suggests that it functions primarily as a signaling regulator rather than a classical engulfment receptor. Studies using TREM2-DAP12-transfected CHO cells demonstrated enhanced uptake of Neuro2A cells without requiring direct ligand recognition, suggesting that TREM2 amplifies intracellular signaling pathways necessary for efficient substrate clearance rather than acting as a conventional scavenger receptor (77, 78). Accordingly, TREM2 cooperates with multiple receptors, including complement receptors, lipid sensors, and scavenger pathways, to establish an appropriate phagocytic response.

In summary, TREM2 functions as a central regulator of microglial surveillance by integrating ligand sensing, intracellular signaling, metabolic adaptation, and intercellular communication. Rather than simply promoting phagocytosis, TREM2 establishes the threshold, timing, and selectivity of microglial remodeling programs. Dysregulation of this surveillance system provides a mechanistic basis for the diverse and sometimes opposing roles of TREM2 across CNS disorders discussed below.

### 3.2 TREM2 in neuroimmune regulation

Beyond its well-established role in phagocytosis, TREM2 functions as a master regulator of neuroimmune homeostasis by coordinating immune activation, resolution, and tissue repair rather than simply promoting or suppressing inflammation. Increasing evidence indicates that TREM2 integrates the intensity and duration of extracellular stimuli with cellular identity, disease stage, and the surrounding microenvironment to orchestrate context-dependent immune responses (79). Following CNS injury or tissue stress, activated glial cells—including microglia and astrocytes—rapidly release cytokines, chemokines, and DAMPs, which reshape the local immune landscape (80). Within this dynamic neuroimmune niche, TREM2 operates as a signaling hub that links immune sensing to adaptive cellular responses, thereby promoting the transition from immune surveillance toward tissue remodeling and restoration of homeostasis.

Rather than acting in isolation, TREM2 participates in an integrated neuroglial communication network. Astrocyte-derived inflammatory mediators can activate TREM2-dependent signaling in microglia and facilitate the recruitment of peripheral monocyte-derived macrophages (74), whereas activated microglia reciprocally regulate astrocytic reactivity through cytokine- and growth factor-mediated signaling. These bidirectional interactions influence astrocytic GFAP expression, process remodeling, extracellular matrix deposition, and glial scar formation following CNS injury. Importantly, these responses are not mediated by distinct signaling machinery but instead utilize the same TREM2–DAP12 receptor complex that governs physiological microglial surveillance under homeostatic conditions. Pathological stimuli therefore primarily redirect downstream transcriptional programs toward injury-responsive immune phenotypes rather than fundamentally altering the core signaling architecture.

At the molecular level, activation of TREM2 induces a coordinated transcriptional program associated with immune resolution and tissue repair, including Lgals1, Lgals3, Il1rn, and Grn, while simultaneously restricting excessive production of inflammatory mediators under appropriate conditions. Accordingly, loss of TREM2 frequently results in elevated expression of iNOS, TNF-α, IL-1β, IL-6, and IFN-γ, whereas restoration or enhancement of TREM2 signaling can attenuate inflammatory responses in multiple experimental settings (74, 81). Nevertheless, accumulating evidence also indicates that TREM2 may facilitate selected immune activation programs during specific pathological contexts. These apparently divergent observations suggest that TREM2 primarily regulates the organization, magnitude, and temporal dynamics of neuroimmune responses rather than functioning as a purely pro- or anti-inflammatory receptor.

Emerging evidence further expands the neuroimmune functions of TREM2 beyond innate immunity. Although TREM2 is predominantly expressed in microglia under physiological conditions, recent studies suggest that its downstream signaling network can influence adaptive immune responses both directly and indirectly, particularly through regulation of cytokine production, antigen presentation, and microglia–lymphocyte communication. Consequently, TREM2 contributes to the coordination of innate and adaptive immunity within the CNS, thereby extending its regulatory influence beyond resident myeloid cells.

Of particular importance, neuroimmune regulation mediated by TREM2 is closely integrated with cellular metabolism. By coupling inflammatory signaling with lipid sensing, mitochondrial function, lysosomal activity, and cellular bioenergetics, TREM2 enables microglia to dynamically adapt their functional state according to local metabolic demands. This immunometabolic coupling supports the transition from homeostatic microglia to specialized activation states required for efficient debris clearance, tissue repair, and restoration of CNS homeostasis.

Taken together, TREM2 should be regarded as a central coordinator of neuroimmune adaptation rather than a simple inflammatory regulator. Through integrating innate immune signaling, neuroglial communication, adaptive immune modulation, and metabolic reprogramming, TREM2 establishes a unified regulatory framework that maintains CNS immune homeostasis under physiological conditions while orchestrating context-dependent responses to injury and disease. This general regulatory architecture provides the mechanistic basis for the disease-specific functions of TREM2 discussed in the following section.

### 3.3 TREM2 signaling networks in neuroimmune and metabolic regulation

TREM2 functions as a central signaling hub that translates extracellular lipid ligands, DAMPs, and inflammatory cues into coordinated intracellular responses. Rather than acting through a single downstream pathway, TREM2 organizes a hierarchical signaling architecture that governs microglial survival, migration, phagocytosis, immunometabolic adaptation, and stress responses. Consequently, TREM2-dependent signaling provides the molecular framework through which microglia maintain tissue homeostasis under physiological conditions while dynamically adapting to pathological challenges. In this context, TREM2 should be viewed not simply as an immune receptor but as a signaling integrator that couples neuroimmune sensing to metabolic reprogramming.

At the molecular level, TREM2 signaling is initiated through its association with the adaptor proteins DAP12 and DAP10. Ligand engagement induces phosphorylation of the immunoreceptor tyrosine-based activation motif (ITAM) within DAP12, resulting in recruitment and activation of SYK (62, 63). Activated SYK subsequently serves as the central proximal signaling node that coordinates several interconnected downstream pathways under the modulation of Src-family kinases and CSF1R. Among these pathways, Vav2/3 regulates actin cytoskeletal remodeling and phagocytic cup formation, whereas Pyk2 promotes cellular survival and resistance to apoptosis. In parallel, DAP10 recruits phosphoinositide 3-kinase (PI3K), further amplifying downstream signaling to support microglial proliferation, migration, and metabolic adaptation. Together, these interconnected signaling modules integrate extracellular stimuli into coordinated cellular programs governing phagocytosis, chemotaxis, inflammatory responses, and cell fate determination.

Among the downstream signaling modules, the SYK–PLCγ2 axis has emerged as one of the principal pathways linking immune activation to metabolic adaptation. PLCγ2, a phospholipase highly enriched in myeloid cells, functions as a critical signaling node coupling TREM2 activation to intracellular calcium signaling and metabolic regulation (11, 82). Following activation of the TREM2–DAP12–SYK complex, PLCγ2 hydrolyzes phosphatidylinositol-4,5-bisphosphate (PIP2) into inositol trisphosphate (IP3) and diacylglycerol (DAG) (83, 84). IP3 induces intracellular Ca^2+^ mobilization, whereas DAG activates protein kinase C (PKC)-dependent signaling cascades. Collectively, these signaling events regulate cytokine secretion, cytoskeletal remodeling, inflammasome activation, and phagocytic activity (85). Furthermore, PLCγ2-mediated Ca^2+^ signaling modulates NLRP3 inflammasome assembly, thereby integrating TREM2 activation with innate immune responses and inflammatory adaptation.

Significantly, accumulating evidence indicates that the biological significance of TREM2 signaling extends well beyond classical immune regulation. The PI3K–Akt–mTOR and PLCγ2 signaling pathways coordinately regulate lipid uptake, cholesterol trafficking, lysosomal biogenesis, mitochondrial fitness, and cellular bioenergetics, thereby enabling microglia to accommodate the substantial metabolic demands associated with CNS injury and neurodegeneration. Through these coordinated metabolic programs, TREM2 promotes efficient clearance of lipid-rich myelin debris, apoptotic cells, and protein aggregates while simultaneously limiting oxidative stress and metabolic dysfunction. These findings indicate that metabolic adaptation is not a secondary consequence of TREM2 activation but an integral component of its signaling architecture.

At the systems level, this integrated signaling network provides the molecular basis for the transition of homeostatic microglia toward DAM, a specialized activation state characterized by enhanced phagocytosis, lipid metabolism, lysosomal function, and stress resilience. Under physiological conditions, these signaling pathways continuously support immune surveillance and metabolic homeostasis in resting microglia. However, persistent pathological stimuli progressively rewire this conserved signaling architecture, redirecting downstream transcriptional and metabolic programs toward disease-specific microglial phenotypes. Consequently, although the upstream signaling framework remains largely conserved, its biological outputs become highly dependent on disease stage, tissue microenvironment, and the nature of pathological stimuli.

Taken together, TREM2 operates through an integrated neuroimmune–metabolic signaling network rather than through isolated downstream pathways. The DAP12–SYK–PI3K–PLCγ2 signaling axis forms the core intracellular framework that couples extracellular immune sensing to metabolic adaptation, phagocytosis, and cellular reprogramming. This conserved signaling architecture provides the mechanistic foundation upon which TREM2 exerts distinct biological functions across different CNS disorders, as discussed in the following disease-specific sections.

## 4 TREM2 in central nervous system disorders: disease-specific roles and mechanistic insights

### 4.1 TREM2 in epileptogenesis: neuroinflammation, synaptic remodeling, and excitability control

Epilepsy is a chronic neurological disorder characterized by recurrent hypersynchronous neuronal activity and progressive disruption of neural circuit stability (86). Although current antiseizure therapies effectively control seizures in many patients, they rarely prevent epileptogenesis or reverse established network abnormalities, highlighting the need to identify mechanisms that regulate the transition from acute neuronal injury to chronic circuit dysfunction. Pathologically, epilepsy involves excitatory–inhibitory imbalance, interneuron vulnerability, oxidative stress, blood–brain barrier disruption, and maladaptive synaptic remodeling, processes in which microglia serve as critical regulators.

Unlike neurodegenerative disorders dominated by extracellular protein accumulation or intracellular proteotoxic stress, epilepsy primarily represents a disorder of neuronal network instability and injury-induced immune remodeling. Therefore, the functional significance of TREM2 in epilepsy is likely distinct from its roles in AD, PD, or ALS. In this context, TREM2 may function primarily as a regulator of microglial activation thresholds, enabling microglia to transition between inflammatory containment, debris clearance, and synaptic remodeling programs in response to seizure-associated damage (87, 88).

Following seizure-induced neuronal stress, extracellular ATP, oxidized lipids, damaged neuronal membranes, and myelin-derived debris provide endogenous signals that may activate TREM2 on microglia. Ligand engagement induces TREM2 association with DAP12 and subsequent SYK activation, which initiates multiple downstream signaling branches. Among these, the PI3K-Akt pathway represents a major metabolic and survival axis that supports microglial fitness, mitochondrial function, and adaptive responses to oxidative stress (89). Akt-dependent activation of mTOR-CREB-BDNF-associated programs further contributes to neuronal survival and synaptic stabilization after seizure injury. In parallel, MAPK/ERK signaling promotes microglial proliferation and neurotrophic responses (90–92), whereas interaction with TRPM2-dependent calcium signaling may restrict excessive ROS accumulation and excitotoxic inflammatory amplification (93, 94). Importantly, these pathways should not be considered redundant downstream events; rather, they represent coordinated but functionally distinct outputs through which TREM2 integrates metabolic adaptation, inflammatory control, and neuronal circuit protection.

The role of TREM2 in epilepsy is highly dependent on disease stage and the balance between injury resolution and circuit remodeling. During the early phase after seizure onset, TREM2 activation is likely beneficial by promoting removal of damaged cellular components, limiting excessive inflammatory signaling, and restoring microglial metabolic capacity. This protective function is particularly relevant because acute seizure-associated neuronal injury generates abundant lipid-rich debris and danger signals that require efficient microglial surveillance (95). However, persistent TREM2 activation during chronic epileptogenesis may produce different consequences. Sustained engagement of microglial remodeling programs may enhance complement-dependent synaptic elimination, alter excitatory–inhibitory balance, and interfere with physiological synaptic maintenance, thereby potentially contributing to network hyperexcitability (96). Thus, in epilepsy, the therapeutic challenge is not simply increasing or suppressing TREM2 activity, but restoring the appropriate temporal threshold of microglial activation required for injury resolution without promoting maladaptive circuit remodeling.

Mechanistically, TREM2-associated signaling in epilepsy differs from that observed in other CNS disorders. In AD, TREM2 primarily regulates plaque-associated microglial responses, lipid metabolism, and Aβ containment. In PD, TREM2 is closely linked to α-Syn clearance, mitochondrial stress adaptation, and autophagy–lysosomal competence (97). In ALS, TREM2 contributes to progressive DAM transition under conditions of chronic motor neuron degeneration and proteostatic stress. By contrast, in epilepsy, the dominant biological challenge is not accumulation of pathogenic proteins but restoration of neuronal network homeostasis. Therefore, TREM2-mediated microglial responses are mainly involved in controlling inflammatory thresholds, synaptic remodeling, and excitability regulation rather than long-term clearance of pathological aggregates (98, 99).

Emerging human evidence supports the relevance of TREM2-associated microglial dysfunction in epilepsy. Single-cell transcriptomic analyses of drug-resistant epilepsy and human epileptic cortical tissues have identified altered microglial states characterized by inflammatory activation, impaired phagocytic programs, and reduced expression of homeostatic genes including TREM2-associated pathways (100, 101). Human iPSC-derived microglial models further demonstrate that human microglia can respond to neuronal hyperactivity and regulate inflammatory responses, although species-specific differences in microglial transcriptional states and TREM2-dependent programs highlight the limitations of directly translating findings from rodent seizure models (102, 103). Notably, most experimental epilepsy models capture acute or subacute inflammatory responses, whereas human epilepsy represents a chronic condition involving long-term circuit reorganization. Therefore, whether therapeutic manipulation of TREM2 should enhance repair-associated signaling or restrain excessive remodeling remains unresolved and will likely depend on disease stage, seizure burden, and individual microenvironmental characteristics.

**Figure 2:**
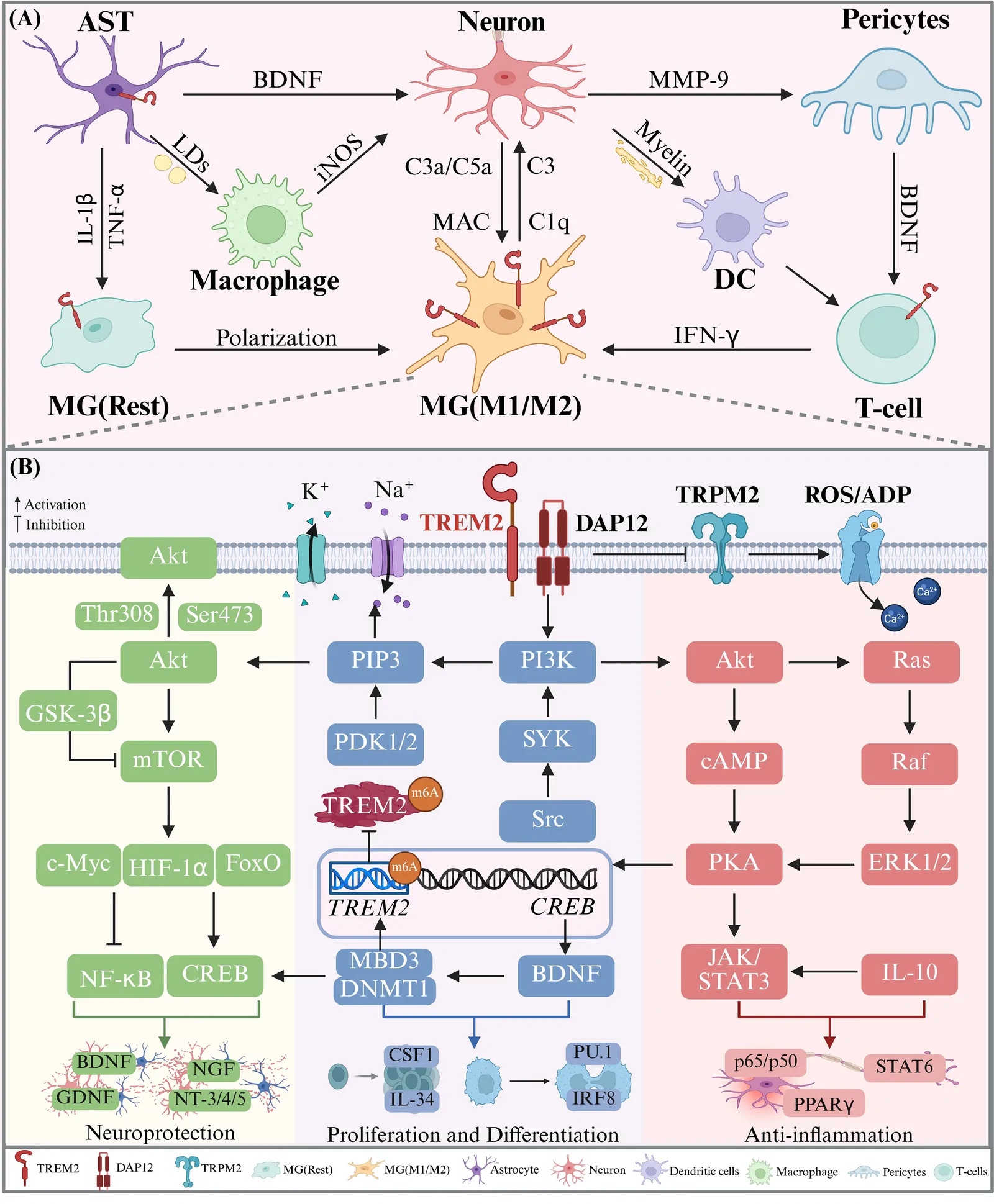
TREM2-centered signaling networks in epilepsy. **(A)** During epileptogenesis, microglial TREM2 functions as a central sensor that integrates damage-associated signals, lipid ligands, and inflammatory cues within the disease-specific neuroimmune microenvironment. Astrocyte-derived IL-1β and TNF-α, lipid droplets, macrophage-derived iNOS, complement components, and intercellular signals involving BDNF, MMP-9, myelin debris, and IFN-γ collectively drive microglial functional remodeling. Through coordinated regulation of phagocytic activity, inflammatory adaptation, and tissue repair, TREM2 contributes to the maintenance of neural circuit homeostasis during epilepsy. **(B)** Upon ligand engagement, TREM2 associates with DAP12 to activate the canonical Src–SYK–PI3K signaling cascade, promoting phosphatidylinositol-3,4,5-trisphosphate (PIP3) generation and subsequent phosphorylation of Akt at Thr308 and Ser473. Downstream activation of the GSK-3β/mTOR pathway regulates c-Myc, HIF-1α, FoxO, NF-κB, and CREB signaling, thereby coordinating ion homeostasis, microglial survival, metabolic adaptation, neurotrophic factor production, proliferation, differentiation, and neuroprotection. Epigenetically, MBD3 recruits DNMT1 to enhance TREM2 promoter methylation, suppressing TREM2 expression and downstream PI3K–Akt signaling, whereas inhibition of the MBD3/DNMT1 axis restores TREM2-dependent protective functions. In parallel, PI3K signaling further integrates with the Akt–cAMP–PKA and Ras–Raf–ERK1/2 pathways, culminating in JAK–STAT3 activation. Together, these interconnected neuroimmune and metabolic signaling networks promote inflammatory resolution, tissue repair, and restoration of neural network homeostasis.

Collectively, TREM2 in epilepsy should be viewed as a dynamic regulator of microglial adaptation rather than a simple pro- or anti-inflammatory molecule. Its therapeutic potential will depend on precisely controlling the transition between protective immune surveillance and maladaptive synaptic remodeling, representing a fundamentally different therapeutic paradigm from TREM2 targeting in protein aggregation-driven neurodegenerative diseases (**Figure 2**).

### 4.2 TREM2 in ischemic stroke: microglial reprogramming, inflammation resolution, and tissue repair

Stroke represents a distinct pathological context for TREM2 biology because, unlike chronic neurodegenerative disorders, it is initiated by acute tissue destruction followed by prolonged phases of inflammation and repair. Secondary neuroinflammation after ischemic injury evolves dynamically from early immune activation to later remodeling processes, with microglia acting as major determinants of both injury propagation and functional recovery (104, 105).

TREM2 has emerged as a central immunometabolic regulator that links damage sensing, lipid metabolism, phagocytic clearance, and inflammatory remodeling in the post-stroke brain (106). Following ischemic injury, endogenous ligands including damaged membrane lipids, myelin debris, and nucleic acid-containing debris activate TREM2 on lesion-associated microglia (107, 108). This activation promotes recruitment of microglia toward injured regions, facilitates debris removal, and supports the transition toward DAM-like states. Importantly, the primary biological demand driving TREM2 activation after stroke differs from that in neurodegenerative diseases: the dominant requirement is rapid containment of tissue damage and restoration of local homeostasis rather than long-term clearance of pathological proteins.

Mechanistically, TREM2-DAP12-SYK signaling provides the proximal signaling platform for cytoskeletal remodeling, migration, and phagocytic adaptation (109). However, TREM2 should not be viewed as a simple anti-inflammatory pathway. Instead, it functions in dynamic interaction with classical injury-associated pathways such as TLR4-p38 MAPK/NF-κB signaling. Whereas TLR4 signaling contributes to rapid inflammatory amplification after ischemic injury, TREM2 signaling promotes debris clearance, metabolic adaptation, and limitation of excessive inflammatory propagation. In parallel, TREM2-associated metabolic programs converge on Nrf2/ARE and SIRT3-dependent mitochondrial pathways, thereby reducing oxidative stress and limiting secondary NLRP3 inflammasome activation.

As ischemic injury progresses, the functional output of TREM2 signaling undergoes temporal transformation. During the early phase, efficient phagocytosis and inflammatory containment dominate, whereas later stages require transition toward regenerative remodeling. TREM2-associated signaling interacts with JAK/STAT pathways, IL-10/TGF-β-mediated immunoregulation, astrocytic responses, and peripheral immune-cell recruitment to shape the balance between inflammation resolution and tissue repair (109–111). Thus, the therapeutic value of TREM2 activation may depend critically on whether signaling occurs during injury containment or during later regenerative phases (112).

Human evidence further highlights this complexity. Elevated plasma sTREM2 levels during acute ischemic stroke have been associated with unfavorable long-term outcomes, including mortality and severe disability (113). Moreover, integrated human-mouse transcriptomic analyses identified a conserved TREM2-GPNMB microglial program associated with poor prognosis (114). These findings raise an important question: whether increased TREM2 expression represents a protective compensatory response that reflects severe injury, or whether persistent TREM2-associated activation contributes directly to maladaptive inflammation (89). Human iPSC-derived microglia studies demonstrate that TREM2 regulates purinergic calcium signaling and migration responses, supporting its potential role in lesion-directed microglial recruitment; however, validation in human post-stroke brain tissue remains necessary (115).

Critically, experimental studies reveal apparently opposing effects of TREM2 after stroke. In middle cerebral artery occlusion (MCAO) models, TREM2 deficiency aggravates ischemic injury by reducing microglial recruitment, impairing debris clearance, and prolonging inflammatory responses (14). Conversely, a photothrombotic stroke model suggested that sustained TREM2 activation may worsen neurological outcomes through prolonged GPNMB-associated DAM-like activation and interference with MANF-mediated regenerative programs (114). These discrepancies likely reflect differences in injury architecture, temporal dynamics, and experimental design rather than intrinsic contradictions.

MCAO produces extensive ischemic necrosis accompanied by substantial debris accumulation, creating a strong demand for TREM2-dependent clearance and metabolic adaptation. Under these conditions, TREM2 activation is predominantly beneficial. In contrast, photothrombotic injury generates localized lesions characterized by persistent remodeling and prolonged microglial activation, where sustained TREM2 signaling may prevent transition from inflammatory containment toward tissue repair (116). Therefore, the ultimate consequence of TREM2 activation depends on whether microglial responses are appropriately terminated after damage clearance or remain chronically engaged (117).

**Figure 3:**
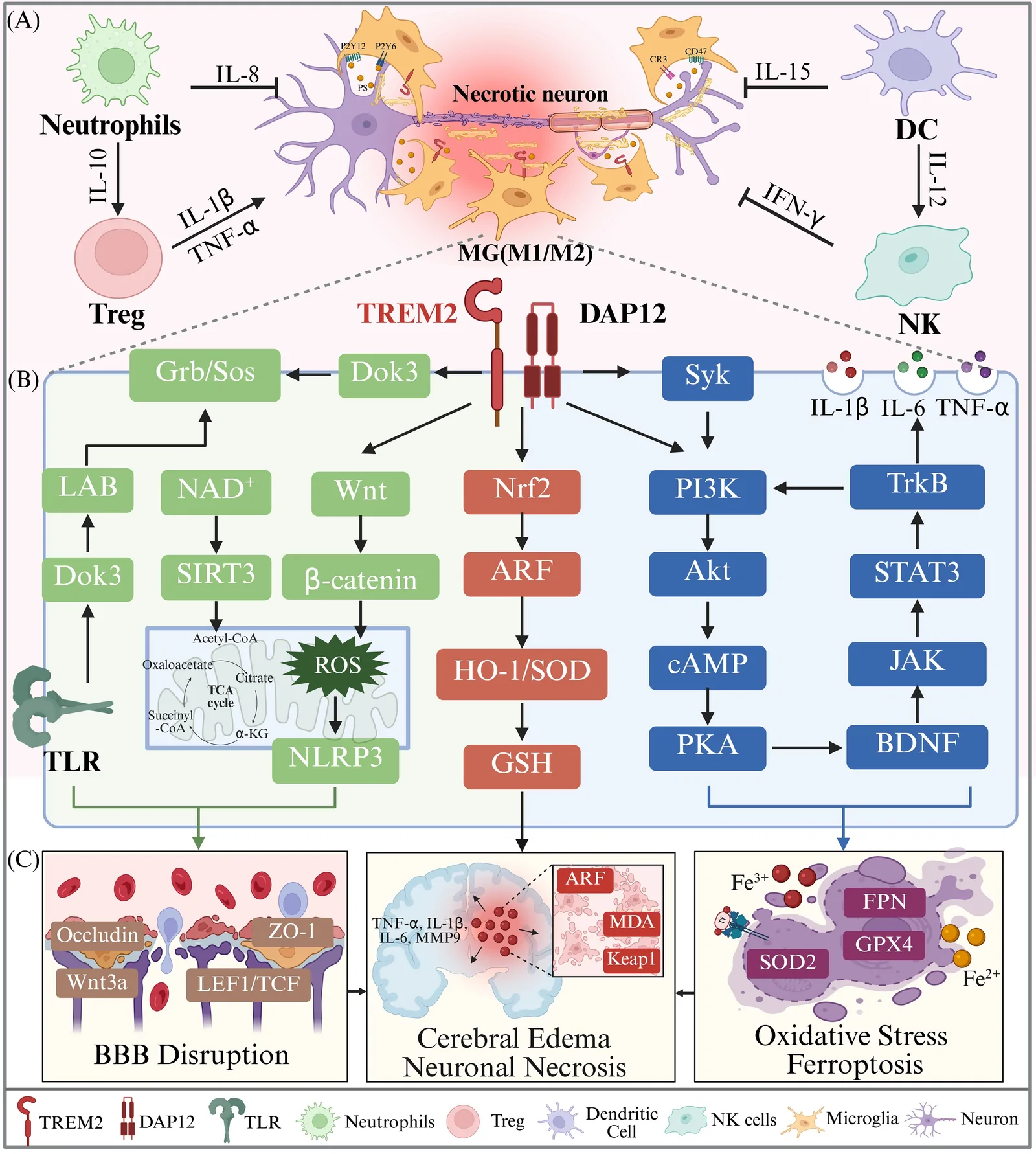
TREM2-mediated neuroimmune regulation and tissue repair after stroke. **(A)** Following ischemic or hemorrhagic stroke, dynamic interactions among neurons, microglia, neutrophils, regulatory T cells, dendritic cells, and natural killer cells establish a disease-specific neuroimmune microenvironment. Cytokines, including IL-1β, TNF-α, and IFN-γ, together with myelin debris and other damage-associated signals, are sensed and integrated by microglial TREM2, which coordinates microglial functional remodeling by balancing inflammatory adaptation with tissue-repair programs. **(B)** Upon ligand engagement, the TREM2–DAP12 complex activates the canonical SYK–PI3K–Akt signaling cascade and its downstream cAMP–PKA pathway while functionally interacting with BDNF–TrkB–JAK–STAT3 signaling. In parallel, TREM2-associated signaling networks involving DOK3/GRB–SOS, NAD^+^/SIRT3, Wnt/β-catenin, and Nrf2/ARE coordinate mitochondrial metabolism, reactive oxygen species production, NLRP3 inflammasome activation, antioxidant defenses, and inflammatory adaptation, thereby integrating neuroimmune responses with metabolic homeostasis during post-stroke repair. **(C)** Through coordinated regulation of neuroimmune and metabolic pathways, TREM2 preserves blood–brain barrier integrity and mitigates oxidative stress and ferroptosis by regulating lipid peroxidation and iron homeostasis, thereby limiting cerebral edema, inflammatory neuronal necrosis, and secondary brain injury. Together, these protective mechanisms promote neurovascular remodeling, tissue repair, and functional recovery after stroke.

Overall, TREM2 functions as a temporal immunometabolic switch after ischemic stroke, coordinating injury containment, inflammatory regulation, and regenerative remodeling. Unlike progressive neurodegenerative diseases, where chronic TREM2 activation may influence DAM states, stroke-associated TREM2 signaling is primarily determined by the transition between acute repair and long-term remodeling. Defining the molecular determinants that convert beneficial TREM2 activation into maladaptive persistence will be essential for developing stage-specific therapeutic strategies (**Figure 3**).

### 4.3 TREM2 in Alzheimer’s disease: amyloid/tau clearance, lipid metabolism, and immune-metabolic reprogramming

Alzheimer’s disease (AD) is the most prevalent neurodegenerative disorder and is neuropathologically characterized by extracellular Aβ plaques and intracellular neurofibrillary tangles composed of hyperphosphorylated tau. Beyond these classical pathological hallmarks, accumulating evidence indicates that impaired lipid metabolism, synaptic dysfunction, and chronic innate immune activation are critical drivers of disease progression. Within this pathological landscape, TREM2 has emerged as a key molecular interface linking physiological microglial surveillance programs with disease-associated immune-metabolic adaptation. Rather than representing a disease-specific pathway, TREM2 signaling in AD appears to reflect a dysregulated reactivation of pre-existing microglial functions. During development, TREM2 contributes to physiological synaptic refinement by regulating the elimination of weak or transient synaptic connections. However, under AD-associated conditions characterized by Aβ accumulation, ApoE-driven lipid imbalance, complement activation, and neuronal stress, this conserved surveillance program may be redirected toward excessive synaptic remodeling (15, 118). Therefore, the pathological consequences of TREM2 activation depend not simply on whether microglial phagocytosis is increased, but on whether the surrounding microenvironment preserves the regulatory mechanisms that normally constrain this process.

A central function of TREM2 in AD is to coordinate microglial adaptation to Aβ pathology (119, 120). Within amyloid-rich niches, ApoE-containing lipoproteins, phosphatidylserine, and Aβ-associated damage signals promote TREM2 clustering and activation, initiating the DAP12-SYK signaling module. This proximal pathway rapidly engages PLCγ2-dependent Ca^2+^ signaling and Vav-Rac1/Cdc42-mediated cytoskeletal remodeling, thereby facilitating microglial migration, plaque enclosure, and phagocytic uptake (121, 122). In parallel, PI3K-AKT-mTOR signaling provides a slower adaptive response by maintaining mitochondrial fitness, lysosomal activity, and lipid-processing capacity during prolonged exposure to lipid-rich pathological substrates. This hierarchical organization suggests that TREM2 does not primarily function as an inflammatory activator, but rather as a metabolic and functional adaptation receptor that enables microglia to cope with sustained pathological stress (123). Consequently, reduced inflammatory marker expression after TREM2 deficiency should not be interpreted as evidence of improved disease outcome, because attenuated cytokine production may coexist with impaired plaque compaction, defective lipid handling, reduced phagocytic competence, and failure to transition toward DAM states.

Notably, the biological consequences of TREM2 activation vary substantially depending on the dominant pathological substrate. In Aβ-driven models, TREM2-mediated microglial recruitment and lipid metabolic adaptation are generally considered protective by promoting plaque containment and limiting extracellular amyloid toxicity. However, whether the same mechanism remains beneficial during tau-driven neurodegeneration remains controversial. Studies using P301S tau models have shown that TREM2 deficiency reduces microglial activation and attenuates tau-associated neuronal loss, suggesting that excessive TREM2-dependent microglial activation may contribute to disease progression (124, 125). Conversely, other studies indicate that loss of TREM2 aggravates tau pathology through impaired microglial adaptation and altered kinase signaling. These apparently contradictory findings likely arise from differences in pathological substrate, disease stage, genetic background, and experimental manipulation strategies. Unlike extracellular Aβ deposition, which requires efficient immune containment and lipid clearance, tau pathology is primarily associated with intracellular neuronal stress and trans-synaptic propagation (126). Under such conditions, prolonged microglial activation may shift from adaptive remodeling toward chronic inflammatory amplification (122). Therefore, TREM2 should not be considered intrinsically protective or detrimental in AD; rather, its functional outcome is determined by the interaction between pathological substrate, microglial state, and temporal disease evolution (127).

Beyond plaque-associated responses, TREM2 serves as a critical regulator of lipid metabolic remodeling in AD-associated microglia. By sensing lipid ligands including ApoE-containing particles, phospholipids, and myelin-derived debris, TREM2 promotes the transition of homeostatic microglia toward DAM states characterized by enhanced lipid uptake, cholesterol trafficking, lysosomal activation, and metabolic adaptation. This process involves coordinated regulation of ApoE-TREM2 signaling (128, 129), PPARγ-dependent lipid metabolism, HIF-1α-associated metabolic adaptation, and complement pathways. At the synaptic level, neuronal stress induces phosphatidylserine exposure, providing an “eat-me” signal recognized by TREM2 and triggering DAP12-SYK-dependent engulfment (130). Complement-dependent mechanisms, including C1q/C3-mediated synaptic tagging, intersect with TREM2 signaling to regulate the threshold of microglial synaptic elimination (131). Under physiological conditions, this coordination ensures selective removal of damaged synapses while preserving functional connectivity. However, in AD, persistent complement activation, lipid overload, and inflammatory stress may lower this threshold and transform an adaptive remodeling program into pathological synaptic loss.

The disease relevance of TREM2 signaling is further shaped by interactions with other cellular compartments. Recent studies indicate that astrocyte-derived signals, including IL-3, can enhance TREM2-dependent microglial responsiveness, whereas infiltrating immune cells modulate microglial activation through cytokine-mediated mechanisms (132). These findings position TREM2 within a broader neuroimmune network involving microglia, astrocytes, neurons, and peripheral immune populations rather than as an isolated microglial receptor (132). Such multicellular interactions may partly explain why TREM2 manipulation produces heterogeneous effects across experimental models.

Importantly, increasing human evidence supports the translational relevance of TREM2 biology while also highlighting limitations of current animal models. Human genetic studies have established rare variants such as R47H, R62H, and H157Y as strong risk factors for AD (133–135), demonstrating that impaired TREM2 function compromises microglial adaptation to pathological stress. Human single-cell transcriptomic and spatial profiling studies further reveal that TREM2 expression is enriched in DAM populations surrounding amyloid pathology, although the functional consequences of these states remain debated. Human iPSC-derived microglial models have demonstrated that TREM2 regulates synaptic recognition and engulfment under AD-related stress conditions, providing mechanistic evidence that complements animal studies (32, 136). However, differences between mouse and human microglial biology, including transcriptional programs, lipid metabolism, and inflammatory responses (38, 137), limit direct extrapolation from experimental models. Moreover, longitudinal biomarker studies indicate that sTREM2 changes dynamically during AD progression and correlates with other pathological biomarkers (138, 139), suggesting that TREM2 activity may represent a temporal process rather than a static disease marker (140, 141).

**Figure 4:**
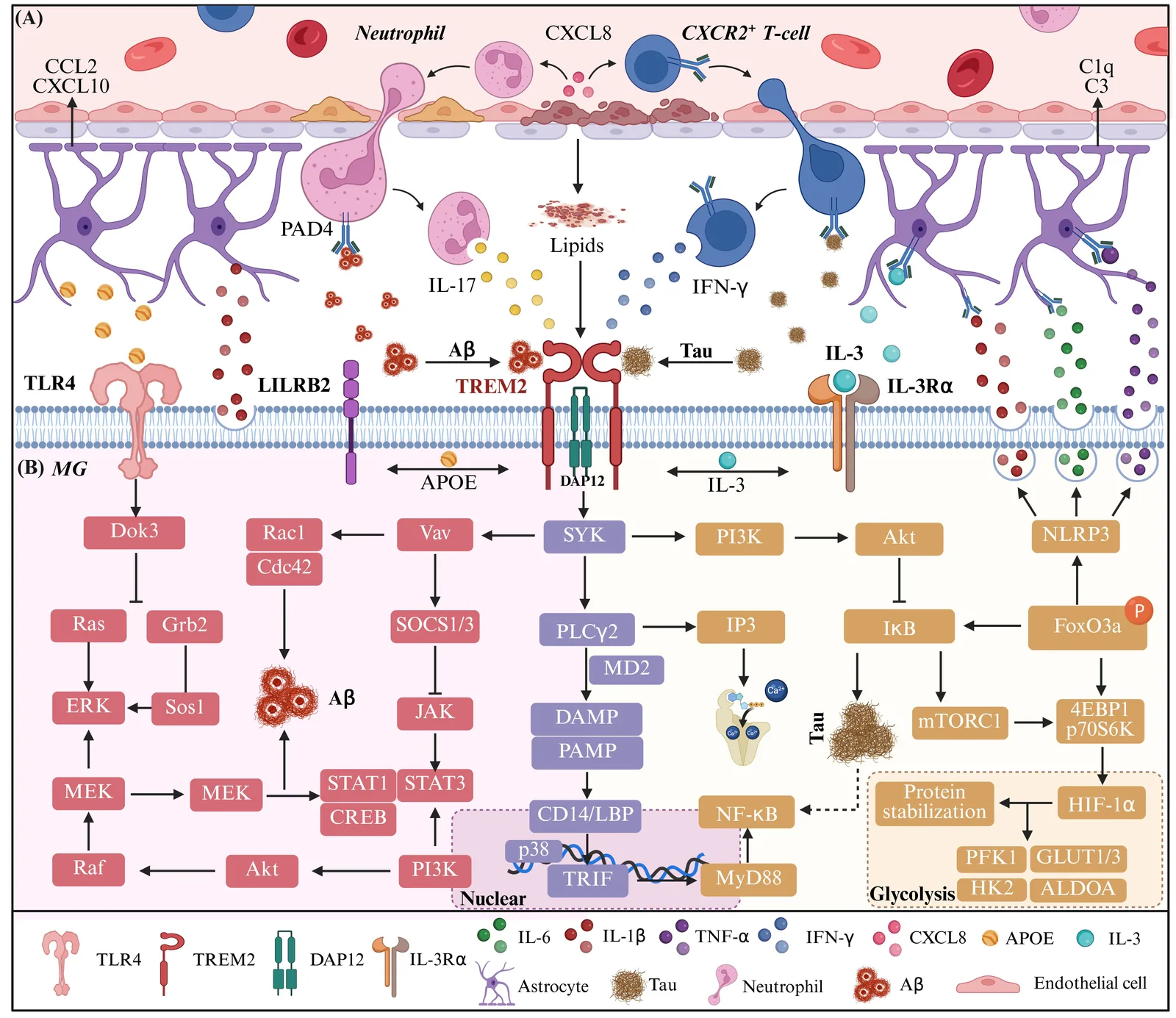
TREM2-driven neuroimmune-metabolic crosstalk in AD. **(A)** In Alzheimer’s disease (AD), BBB dysfunction facilitates the recruitment of neutrophils and CXCR2^+^ T cells in response to chemokines, including CCL2, CXCL10, and CXCL8, thereby establishing a disease-specific neuroimmune microenvironment. Neutrophil-derived IL-17, lymphocyte-derived IFN-γ, astrocyte-derived inflammatory mediators, and complement components cooperate with Aβ, tau, lipids, APOE, and IL-3–IL-3Rα signaling to converge on TREM2-expressing microglia, driving their transition toward DAM and coordinating pathological protein clearance, lipid metabolism, and immunometabolic adaptation. **(B)** Upon ligand engagement, the TREM2–DAP12 complex recruits SYK and activates the canonical PLCγ2–IP3–Ca^2+^, Vav–Rac1/Cdc42, and PI3K–Akt signaling cascades, thereby regulating calcium homeostasis, cytoskeletal remodeling, phagocytosis, cell survival, lipid metabolism, and metabolic reprogramming. Downstream PI3K–Akt signaling further modulates the IκB–NF-κB, FoxO3a–NLRP3, and mTORC1–HIF-1α pathways to coordinate inflammatory adaptation, protein homeostasis, and glycolytic remodeling. In parallel, TREM2-associated Vav–SOCS1/3 and PLCγ2 signaling functionally interact with the TLR4–MEK–ERK and JAK–STAT pathways, facilitating Aβ and tau clearance while limiting excessive inflammatory activation. Together, these interconnected neuroimmune and metabolic signaling networks promote microglial adaptation, preserve cellular metabolic homeostasis, and mitigate proteinopathy, neuroinflammation, and neuronal injury during Alzheimer’s disease progression.

These observations have important therapeutic implications (142). Although enhancement of TREM2 signaling has shown promise in amyloid-centered models, broad and persistent activation may not represent an optimal strategy for human AD, particularly in later disease stages dominated by tau pathology and chronic inflammatory remodeling. Future therapeutic approaches will likely require biomarker-guided and stage-specific modulation of TREM2 activity (139, 143), aiming not simply to increase receptor activation but to restore the balance between microglial clearance capacity, lipid metabolic homeostasis, and inflammatory restraint. Thus, TREM2 represents neither a universal protective factor nor a pathogenic driver in AD; rather, it functions as a dynamic immunometabolic regulator whose biological effects are determined by disease stage, pathological substrate, and cellular context (144) (**Figure 4**).

### 4.4 TREM2 in Parkinson’s disease: α-synuclein pathology, autophagy-inflammation coupling, and dopaminergic neurodegeneration

Parkinson’s disease (PD) is the second most common neurodegenerative disorder, characterized by progressive degeneration of dopaminergic neurons in the substantia nigra pars compacta (SNpc), accumulation of misfolded α-Syn, mitochondrial dysfunction, and chronic neuroimmune activation (145). Although α-Syn aggregation has traditionally been considered the primary pathological hallmark of PD, increasing evidence indicates that impaired proteostasis, defective lysosomal degradation, altered lipid metabolism, and maladaptive microglial responses collectively determine neuronal vulnerability. Given the high metabolic demands and limited regenerative capacity of dopaminergic neurons, microglial dysfunction may represent a critical amplifier linking α-Syn accumulation, oxidative stress, and progressive neurodegeneration.

Emerging evidence suggests that TREM2 functions as an immunometabolic regulator that connects α-Syn handling, lysosomal adaptation, and inflammatory remodeling in PD (146, 147). Unlike AD, where TREM2 signaling is largely associated with extracellular Aβ plaque recognition and lipid-rich lesion containment, the major pathological challenge faced by TREM2-positive microglia in PD is the continuous processing of intracellularly derived α-Syn species and damaged organelles. Following exposure to α-Syn aggregates, lipid-associated stress signals, or neuronal injury-associated ligands, TREM2 recruits the adaptor protein DAP12 and activates SYK, which serves as a central signaling hub integrating multiple downstream programs. Among these pathways, the PI3K-AKT-mTOR axis appears particularly important for maintaining microglial metabolic fitness, survival, and lysosomal competence, thereby sustaining the capacity required for α-Syn uptake and degradation (148). Thus, the primary function of TREM2 signaling in PD may not simply be to enhance inflammatory activation, but rather to preserve microglial functional competence under persistent proteostatic stress.

Downstream of SYK activation, TREM2 signaling undergoes hierarchical divergence into several functionally distinct branches. The PI3K-AKT-mTOR pathway provides long-term metabolic support by regulating mitochondrial activity, lipid utilization, and autophagy-related processes, whereas ERK/TFEB signaling promotes lysosomal biogenesis and clearance capacity. In parallel, Src-associated signaling can influence NF-κB-dependent inflammatory programs and STAT3-mediated survival responses. Therefore, the biological consequence of TREM2 activation is determined not by a single pathway but by the relative balance among inflammatory activation, metabolic adaptation, and proteostatic maintenance. This signaling hierarchy provides a mechanistic explanation for the apparently contradictory observations that TREM2 deficiency may reduce inflammatory markers while simultaneously accelerating neurodegeneration.

Indeed, in acute toxin-induced PD models such as MPTP, TREM2 deficiency decreases the expression of inflammatory mediators including TNF-α, IL-1β, and iNOS, suggesting reduced microglial inflammatory activation. However, MPTP exposure strongly activates the TLR4-TRAF6-MAPK/NF-κB inflammatory cascade, and the reduction of cytokine production following TREM2 loss may therefore represent suppression of an acute inflammatory response rather than restoration of microglial homeostasis. Importantly, this inflammatory attenuation is accompanied by impaired phagocytic adaptation, defective lysosomal function, reduced metabolic flexibility, and compromised α-Syn clearance capacity (36). These findings indicate that decreased inflammatory signaling should not be interpreted as equivalent to neuroprotection, because microglia may enter a dysfunctional hypo-responsive state characterized by reduced immune activity but impaired tissue maintenance.

Conversely, enhanced TREM2 activation appears capable of limiting excessive inflammatory amplification under specific pathological conditions. TREM2 overexpression promotes a reparative microglial phenotype and increases responsiveness to IL-4/IL-13-mediated anti-inflammatory programs through STAT-dependent mechanisms, thereby reducing α-Syn-induced neurotoxicity (149). Mechanistically, TREM2 also interacts with the TLR4-TRAF6 pathway, which is strongly activated during α-Syn-induced inflammatory stress (150). TREM2 signaling suppresses excessive TLR4/TRAF6 activation and downstream p38, JNK, ERK, NF-κB, and NLRP3 inflammasome signaling, while simultaneously maintaining lysosomal and autophagic capacity through TFEB and mTOR-associated pathways (151–153). Thus, TREM2 does not function as a simple inflammatory activator or inhibitor; rather, it acts as a buffering system that restrains uncontrolled inflammation while sustaining the cellular machinery required for pathological substrate clearance.

The stage-dependent nature of TREM2 signaling is particularly relevant in PD progression. During early disease stages, when α-Syn accumulation and mitochondrial stress remain within the adaptive capacity of microglia, TREM2-mediated metabolic reprogramming, lipid processing, lysosomal activation, and phagocytic responses may promote neuronal protection. However, with prolonged exposure to α-Syn aggregates, oxidative stress, and mitochondrial dysfunction, persistent demand for TREM2-dependent adaptation may exceed microglial capacity, resulting in chronic inflammatory remodeling and failure of effective proteostasis (154). Therefore, the detrimental effects associated with sustained TREM2 activation may not reflect an inherently pathogenic function of TREM2, but rather the inability of an initially protective adaptive program to resolve persistent pathological stress.

Beyond intrinsic microglial responses, TREM2 also regulates neuron–glia and glia–glia communication in PD. Activated microglia release IL-1α, TNF-α, and C1q, which contribute to the induction of neurotoxic A1 astrocytes and amplify inflammatory feedback loops (146). TREM2 may modulate this microglia–astrocyte interaction by controlling the threshold and duration of microglial activation. Under physiological conditions or early pathological stages, TREM2 maintains microglial surveillance, lipid metabolism, and lysosomal homeostasis, functions that are particularly important for protecting dopaminergic neurons that are highly vulnerable to oxidative stress and impaired α-Syn clearance. However, under persistent inflammatory stimulation, dysregulated TREM2-associated programs may contribute to maladaptive glial remodeling rather than effective repair (155).

Importantly, human studies increasingly support the relevance of TREM2-associated microglial remodeling in PD. Single-nucleus RNA sequencing analyses of post-mortem human midbrain have revealed a transition from P2RY12-high homeostatic microglia toward stress-associated states enriched in GPNMB, IL1B, and HSP90AA1, indicating DAM transformation in PD (113). Furthermore, elevated CSF sTREM2 has been preferentially observed in PD patients with tau-positive biomarker profiles, suggesting that TREM2 activation may reflect specific neuronal injury contexts rather than representing a universal feature of PD progression (114). These human observations emphasize the importance of interpreting TREM2 activity within specific pathological environments rather than assuming uniform effects across patients.

**Figure 5:**
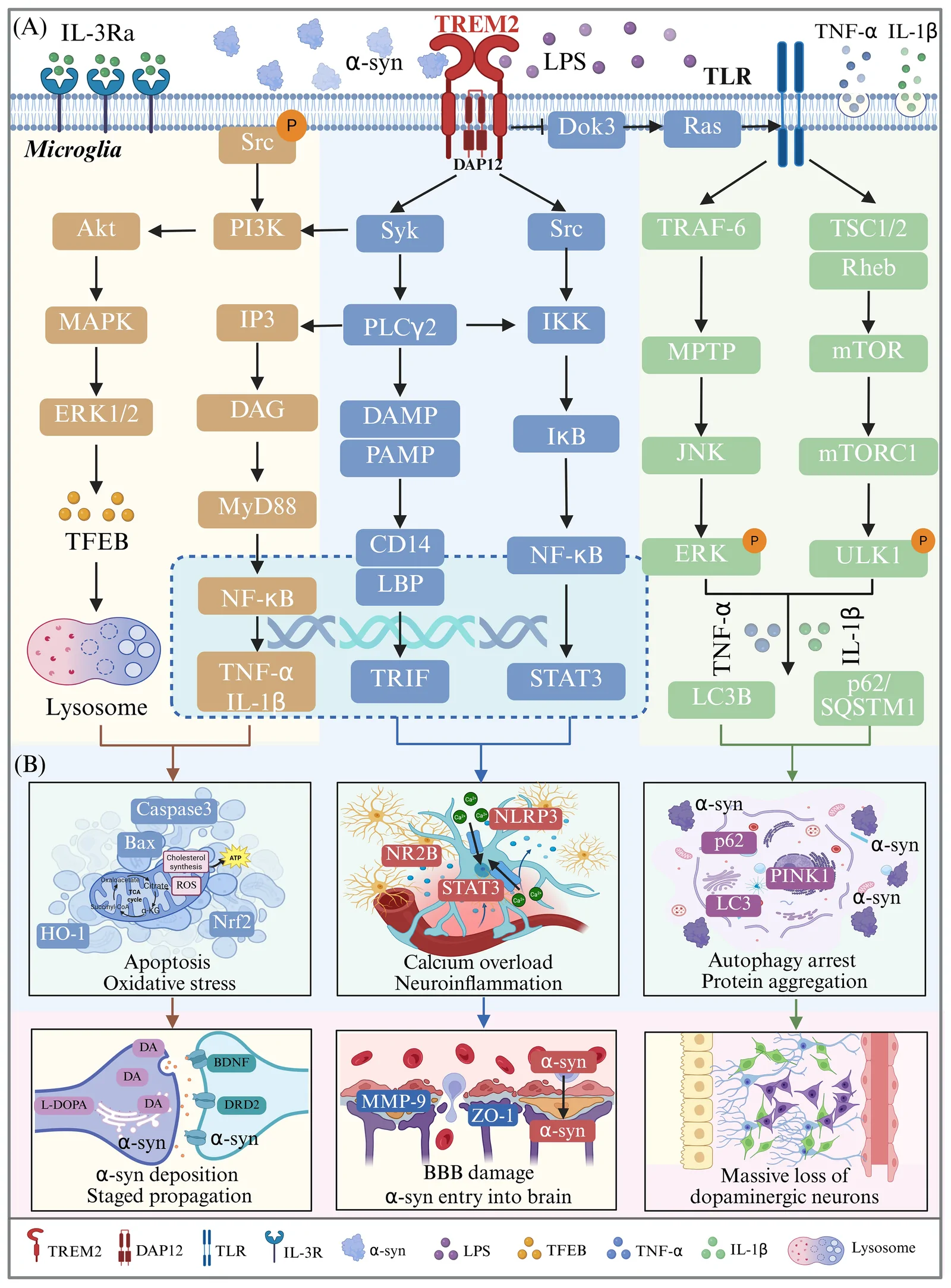
TREM2-mediated neuroimmune and autophagy signaling networks in PD. **(A)** In Parkinson’s disease (PD), extracellular α-synuclein (α-syn) aggregates and lipopolysaccharide-associated inflammatory stimuli establish a disease-specific neuroimmune microenvironment that drives microglial functional remodeling. Acting as a central sensor of proteotoxic and inflammatory stress, TREM2 integrates α-syn- and pathogen-associated signals through DAP12-, Toll-like receptor-, and IL-3Rα-associated pathways to coordinate inflammatory adaptation, lysosomal function, and immunometabolic homeostasis. Upon ligand engagement, the TREM2–DAP12 complex recruits SYK and activates the canonical PLCγ2–IP3–Ca^2+^, PI3K–Akt–MAPK/ERK, and Src–IKK–IκB–NF-κB signaling cascades, thereby regulating calcium homeostasis, inflammatory gene expression, microglial survival, and metabolic adaptation. PI3K–Akt–ERK signaling further activates TFEB-dependent lysosomal programs, whereas PLCγ2-associated DAMP/PAMP sensing functionally interfaces with CD14/LBP–TRIF/MyD88 signaling. In parallel, crosstalk between TREM2 and Toll-like receptor signaling engages the TRAF6–JNK/ERK and mTOR–mTORC1–ULK1 pathways to regulate TNF-α and IL-1β production as well as lysosomal-autophagic activity. **(B)** Dysregulation of these interconnected neuroimmune and metabolic signaling pathways promotes mitochondrial dysfunction, oxidative stress, apoptosis, calcium overload, NLRP3 inflammasome activation, and lysosomal-autophagy dysfunction. These pathological alterations impair α-syn clearance, facilitate α-syn aggregation and propagation, compromise BBB integrity, and accelerate progressive dopaminergic neuronal degeneration. Conversely, appropriate TREM2 signaling enhances lysosomal and autophagic clearance of α-syn, restores immunometabolic homeostasis, limits excessive inflammatory and oxidative injury, and preserves dopaminergic neuronal integrity during PD progression.

In summary, TREM2 represents a stage- and context-dependent immunometabolic regulator in PD that integrates α-Syn clearance, lysosomal-autophagic adaptation, inflammatory regulation, and neuron–glia communication. The apparent paradox that TREM2 deficiency suppresses inflammatory markers yet aggravates neurodegeneration highlights a fundamental principle of microglial biology: inflammatory intensity does not necessarily reflect functional immune competence. Future therapeutic strategies should therefore move beyond simple TREM2 activation or inhibition and instead aim to restore disease-stage-specific balance among microglial clearance capacity, metabolic fitness, and inflammatory resolution (**Figure 5**).

### 4.5 TREM2 in amyotrophic lateral sclerosis: microglial state transition, proteostasis, and neuroinflammatory control

Amyotrophic lateral sclerosis (ALS) is a rapidly progressive neurodegenerative disorder characterized by selective degeneration of upper and lower motor neurons in the motor cortex and spinal cord, resulting in progressive muscle weakness, paralysis, and respiratory failure (156). Although a subset of ALS cases is associated with mutations in genes including SOD1, TARDBP, and C9orf72, the majority of cases remain sporadic and lack effective disease-modifying therapies despite the availability of riluzole and edaravone (157). Unlike AD and PD, where extracellular Aβ deposition or α-Syn accumulation represents a relatively defined pathological trigger, ALS is characterized by continuous motor neuron injury, axonal degeneration, and intracellular proteostatic disruption. These unique pathological features create a chronic neuroimmune environment in which microglia must continuously adapt to persistent neuronal stress rather than respond to a single pathological substrate. Therefore, TREM2 may primarily function in ALS as a regulator of microglial state transition, metabolic adaptation, and long-term tissue surveillance.

Increasing evidence indicates that ALS is not solely a neuron-autonomous disorder but is critically shaped by dynamic interactions among motor neurons, microglia, astrocytes, and peripheral immune components (104). In this context, TREM2 has emerged as an important regulator of DAM remodeling. Unlike the classical M1/M2 polarization paradigm, ALS-associated microglia exist along a transcriptional continuum characterized by progressive alterations in lipid metabolism, phagocytic activity, inflammatory signaling, and stress responses (158). TREM2, together with ApoE and TGF-β-associated pathways (159), facilitates the transition from homeostatic microglia toward disease-associated states, suggesting that it functions as a molecular switch enabling microglia to adapt to chronic motor neuron-derived stress signals.

In SOD1- and TDP-43-based ALS models, TREM2 deficiency markedly impairs the induction of DAM-related transcriptional programs (160), resulting in partial maintenance of homeostatic signatures and reduced adaptive responses (161, 162). Recent studies further indicate that DAM activation occurs through a multistep transition, in which TREM2-dependent intermediate states precede full DAM conversion (108). This finding suggests that TREM2 does not simply activate a fixed inflammatory phenotype but enables progressive microglial adaptation to increasing pathological demands. During early ALS progression, TREM2-dependent remodeling may enhance lipid utilization, lysosomal activity, phagocytic capacity, and metabolic resilience, thereby supporting tissue compensation. However, as motor neuron degeneration persists and intracellular protein stress accumulates, the metabolic burden required to sustain these adaptive programs may exceed microglial capacity, resulting in impaired metabolic flexibility, defective clearance responses, and persistent inflammatory remodeling. Thus, the detrimental consequences associated with chronic TREM2 activation may represent an exhausted or maladaptive adaptation rather than an intrinsically pathogenic function of TREM2 signaling.

A key distinction between ALS and other neurodegenerative disorders is the intracellular nature and continuous production of pathological proteins, particularly TDP-43 aggregates (156). Unlike Aβ plaques in AD, which accumulate extracellularly and require plaque-associated containment, TDP-43 pathology primarily reflects intracellular disruption of neuronal proteostasis and progressive cellular degeneration. Consequently, TREM2-mediated microglial responses in ALS may contribute less to direct aggregate clearance and more to maintaining a functional immune-metabolic environment capable of responding to ongoing neuronal stress. In TDP-43-driven neurodegeneration, loss of TREM2 impairs microglial phagocytic activity and accelerates neuronal injury and motor dysfunction (163, 164). Moreover, TREM2 regulates the transition of microglia away from a homeostatic state by controlling the downregulation of homeostatic markers such as P2RY12 and TMEM119 during disease progression. Therefore, TREM2 appears to be required not only for substrate clearance but also for enabling microglia to undergo appropriate functional state switching in response to chronic neurodegenerative stress.

The functional importance of TREM2 in ALS is further linked to its interaction with the CSF1R-DAP12 signaling network, which regulates microglial survival, proliferation, and regional persistence (165). Because ALS involves progressive and spatially expanding motor neuron degeneration, sustained microglial maintenance within vulnerable regions represents a critical biological requirement. Through shared DAP12-dependent signaling, TREM2 and CSF1R cooperate to regulate SYK activation, PLCγ2 signaling, CREB-dependent survival pathways, and metabolic adaptation. This signaling architecture differs fundamentally from its role in AD and PD. In AD, TREM2-associated signaling primarily supports plaque-associated microglial organization and lipid buffering in response to extracellular Aβ and ApoE-rich environments. In PD, the major challenge is persistent α-Syn accumulation and mitochondrial dysfunction, making TREM2-dependent autophagy-lysosomal coupling and proteostatic maintenance particularly important. In contrast, ALS is characterized by continuous motor neuron degeneration and intracellular proteostatic stress, in which TREM2 primarily regulates microglial state transition, survival, and long-term adaptation to lesion persistence. Therefore, although AD, PD, and ALS share the conserved TREM2-DAP12-SYK-PI3K-AKT/MAPK-NF-κB signaling framework, the biological output of this pathway is determined by the dominant pathological substrate and cellular demand: plaque-interface regulation in AD, autophagy-proteostasis maintenance in PD, and adaptive state transition in ALS.

Human studies further support the clinical relevance of TREM2-associated microglial remodeling in ALS while highlighting substantial disease heterogeneity. Single-nucleus RNA sequencing analyses of human motor cortex and spinal cord, combined with patient-derived iPSC microglial models, have demonstrated distinct microglial responses among ALS subtypes, with enhanced disease-associated activation in sporadic ALS but attenuated activation and altered endolysosomal programs in C9orf72-associated ALS (166). Increased TREM2 expression in post-mortem spinal cord tissue and elevated CSF and serum sTREM2 levels further indicate activation of the TREM2 pathway during human ALS progression (9). However, whether these alterations represent protective compensation or pathogenic amplification remains unresolved, emphasizing the importance of interpreting TREM2 activation within specific genetic and cellular contexts.

Genetic studies further suggest that impaired TREM2 function may influence ALS susceptibility and progression. Variants including R47H, R62H, and D87N have been associated with altered risk profiles, although their functional consequences in ALS remain less well defined than in AD. These variants may impair ligand recognition, downstream signaling efficiency, or microglial adaptive capacity, thereby reducing the ability of microglia to respond appropriately to chronic neurodegenerative stress. In parallel, widespread immune transcriptomic alterations observed in ALS motor cortex support the concept that innate immune dysregulation contributes to disease heterogeneity.

Beyond membrane-bound TREM2, sTREM2 has emerged as a potential biomarker and functional regulator in ALS. Increased CSF sTREM2 levels have been detected in ALS patients, particularly during early disease stages, and higher levels have been associated with slower disease progression, suggesting a potential compensatory role (167). Experimental studies further indicate that sTREM2 may promote microglial survival and enhance responses to pathological substrates, although whether these effects are mediated through classical TREM2 signaling pathways or independent mechanisms remains unclear.

Collectively, TREM2 functions as a central regulator of microglial adaptation in ALS by integrating motor neuron-derived stress signals, proteostatic disruption, metabolic remodeling, and inflammatory regulation. Unlike AD and PD, where TREM2 activity is primarily interpreted through the lens of plaque containment or α-Syn clearance, ALS highlights the role of TREM2 in sustaining microglial state flexibility during prolonged neuronal degeneration. Thus, TREM2 should not be considered a simple protective or detrimental factor but rather a dynamic regulator whose effects depend on disease stage, genetic background, pathological burden, and microglial adaptive capacity. Future therapeutic approaches should therefore focus on restoring appropriate TREM2-mediated microglial plasticity rather than indiscriminate activation, with the goal of preserving adaptive responses while preventing chronic maladaptive immune remodeling.

## 5 Clinical application of TREM2 as a therapeutic target

**Figure 6:**
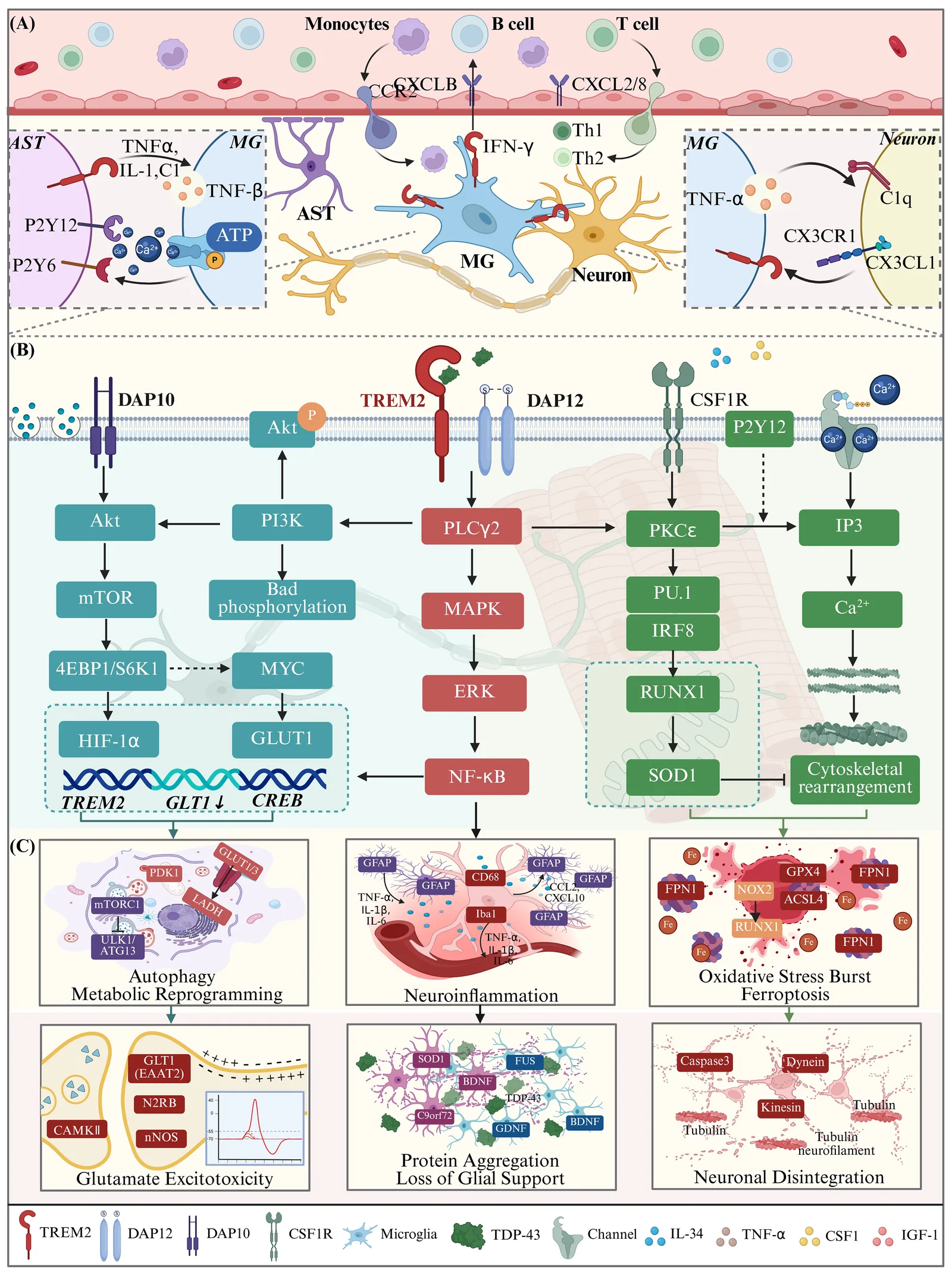
TREM2-dependent microglial reprogramming in ALS. **(A)** In amyotrophic lateral sclerosis (ALS), bidirectional communication among microglia, astrocytes, neurons, and infiltrating peripheral immune cells establishes a disease-specific neuroimmune microenvironment. Following blood–brain barrier disruption, peripheral immune cells, including B cells and T cells, infiltrate the central nervous system in response to endothelial-derived chemokines, including CXCL8 and CXCL2, thereby amplifying neuroimmune interactions. Acting as a central sensor of neuronal injury and proteotoxic stress, TREM2 integrates pathological protein- and immune-derived signals to drive microglial functional remodeling and promote the transition toward disease-associated microglial phenotypes. **(B)** Upon ligand engagement, the TREM2–DAP12 complex activates the canonical PLCγ2, PI3K–Akt, and MAPK–ERK–NF-κB signaling pathways. In parallel, DAP10-dependent Akt–mTOR–4EBP1/S6K1 signaling regulates MYC, HIF-1α, GLUT1, and related transcriptional programs to coordinate microglial survival, metabolic adaptation, and immunometabolic reprogramming. TREM2 signaling further functionally interfaces with the CSF1R–RUNX1–SOD1 axis and P2Y12–IP3–Ca^2+^ signaling, thereby regulating antioxidant defenses, cytoskeletal remodeling, phagocytic activity, and microglial stress adaptation. **(C)** These interconnected neuroimmune and metabolic signaling networks regulate autophagy, metabolic reprogramming, oxidative stress, ferroptosis, glutamate excitotoxicity, pathological protein accumulation, and loss of glial support. By restoring microglial homeostasis and limiting these disease-associated pathological processes, TREM2 signaling may preserve motor-neuron integrity and slow ALS progression.

TREM2 has emerged as a promising therapeutic target for CNS disorders by regulating microglial survival, phagocytic competence, lipid metabolism, and inflammatory adaptation. However, accumulating evidence indicates that TREM2 does not function as an intrinsically protective or detrimental pathway; rather, its biological consequences are determined by disease stage, pathological substrate, microenvironmental signals, and the functional state of microglia. Therefore, therapeutic strategies targeting TREM2 should not be designed simply to maximize receptor activation, but instead aim to restore appropriate TREM2-dependent microglial adaptability. This consideration is particularly important in chronic neurodegenerative disorders, in which microglia may transition from adaptive responders during early disease stages to metabolically exhausted or maladaptive states during disease progression (**Figure 6**).

### 5.1 TREM2 agonists and clinical development

Current approaches aimed at enhancing TREM2 signaling include agonistic antibodies, endogenous lipid-associated ligands, peptide-based modulators, and strategies regulating receptor stability. Candidate molecules include HSP60, ApoE-derived ligands, AL002a (168), COG1410 (96), and 4D9 (169). Among these approaches, agonistic antibodies represent the most clinically advanced strategy because they provide relatively selective modulation of membrane-bound TREM2 signaling while avoiding broad activation of other myeloid immune receptors (170). However, the therapeutic rationale for TREM2 agonism requires careful interpretation, as receptor activation may produce distinct outcomes depending on the pathological environment in which microglia operate.

The 4D9 antibody represents an alternative strategy that increases membrane-associated TREM2 availability by reducing receptor ectodomain shedding through interference with ADAM10/17-mediated cleavage (171). By preserving full-length TREM2, this approach enhances downstream signaling capacity and promotes microglial survival and phagocytic activity in experimental models. Similarly, COG1410, an ApoE-mimetic peptide, and other experimental ligands including AMG520 and HSP60 have been reported to enhance TREM2-dependent microglial responses and reduce inflammatory phenotypes in preclinical studies. Nevertheless, whether these approaches restore functional microglial states in human neurological disorders remains uncertain, particularly because DAM in humans exhibit substantial heterogeneity that cannot be fully reproduced in conventional animal models.

AL002a represents the most clinically advanced TREM2 agonistic antibody developed to date. By engaging the TREM2 stalk region, AL002 enhances receptor clustering and downstream signaling activation. In amyloid-based mouse models, AL002 increased plaque-associated microglial accumulation, promoted microglial proliferation, and reduced neuritic dystrophy surrounding amyloid plaques (168). These findings supported the concept that enhancing TREM2 activity could restore defective microglial plaque containment and clearance capacity during early AD. However, the recent phase II clinical trial of AL002 in patients with early AD failed to demonstrate significant clinical benefit despite confirming target engagement and acceptable safety (172). This outcome highlights a critical translational challenge: effective engagement of a molecular target does not necessarily indicate restoration of disease-modifying microglial function.

Several factors may explain the limited efficacy of TREM2 agonism in clinical settings. First, most preclinical evidence supporting TREM2 activation has been derived from amyloid-centric models, including APP/PS1 and 5xFAD mice, in which plaque-associated microglial recruitment and compaction represent dominant pathological processes. However, human AD is characterized by complex interactions among Aβ deposition, tau propagation, lipid metabolic dysfunction, vascular alterations, and chronic immune remodeling. In particular, TREM2-mediated microglial activation may have distinct consequences in tau-dominant environments, where sustained inflammatory remodeling may become maladaptive rather than protective. Therefore, the therapeutic effect of TREM2 activation observed in amyloid models may not fully predict responses in heterogeneous human AD populations. Second, enhancing TREM2 signaling alone may be insufficient to overcome advanced-stage microglial dysfunction. During disease progression, microglia undergo profound metabolic and epigenetic remodeling, including altered cholesterol metabolism, lysosomal stress, mitochondrial dysfunction, and reduced adaptive capacity (154, 173). Under these conditions, increasing receptor activation may amplify signaling in dysfunctional cells without restoring their ability to execute beneficial functions. Thus, a central challenge for future TREM2-directed therapy is determining whether target microglia retain sufficient functional plasticity to respond to receptor stimulation. Third, therapeutic efficacy may depend on accurate patient stratification and disease-stage selection. Genetic background, including TREM2 variants and ApoE genotype, may influence receptor responsiveness and downstream signaling capacity. Furthermore, dynamic biomarkers such as CSF sTREM2, amyloid/tau profiles, inflammatory signatures, and imaging-based indicators of microglial activation may help identify patients most likely to benefit from specific TREM2-targeted interventions (174). Rather than applying universal TREM2 activation across disease populations, future strategies may require biomarker-guided approaches that match therapeutic modulation to specific pathological states.

Beyond direct receptor agonism, emerging approaches may focus on restoring the broader TREM2 signaling network. Modulating downstream pathways including DAP12-SYK-PLCγ2, PI3K-AKT-mTOR, TFEB-dependent lysosomal programs, and lipid metabolic regulators may provide alternative strategies to enhance microglial function without excessive immune activation. In addition, controlling the balance between membrane-bound TREM2 and sTREM2 represents another potential therapeutic avenue, as sTREM2 may function not only as a biomarker but also as an active regulator of microglial survival and immune adaptation. Such approaches may ultimately allow more precise manipulation of TREM2-associated biology.

Overall, current evidence suggests that successful TREM2-based therapies will depend not on maximizing receptor activity, but on restoring the appropriate balance among microglial clearance capacity, metabolic fitness, and inflammatory regulation. Future clinical translation will require a shift from broad TREM2 activation toward precision immunomodulation that considers disease stage, pathological substrate, genetic background, and microglial functional state. Defining the molecular determinants that distinguish adaptive from maladaptive TREM2 signaling will be essential for developing effective therapeutic strategies across CNS disorders (**Table 2**).

**Table 2:** Representative pharmacological strategies targeting TREM2 signaling: molecular characteristics, mechanisms of action, translational status and disease applications.

Pharmacological strategy	Agent	Molecular type	Molecular weight	Binding region	Mechanism of TREM2 modulation	Development status	Clinical indications	Disease models	References
Direct agonists	HSP60	Protein	52-65KDa	TREM2 extracellular Ig domain	Activation of TREM2-related signaling pathway by binding to immunoglobulin region of TREM2 extracellular domain	Preclinical *in vitro* & rodent	Alzheimer’s disease, ischemic stroke, traumatic brain injury	5XFAD AD mice, mouse cerebral ischemia model, diabetic cognitive impairment mice	(57)
	ApoE	Protein	63-67KDa	Soluble TREM2-Fc domain, C-terminal lipid-binding pocket of ApoE	Residues bind to soluble TREM2-Fc	Preclinical *in vitro* & transgenic mice	AD, cerebral hemorrhage, tauopathy	5XFAD, APP/PS1, P301L tau mice, post-stroke mouse model	(50)
	COG1410	Polypeptide	1408.78Da	TREM2 extracellular domain	An ApoE-derived peptide	Preclinical rodent *in vivo*	Chronic sleep deprivation-induced cognitive decline, traumatic brain injury, AD	Sleep-deprived C57BL/6 mice, TBI mouse model, 5XFAD mice	(96)
	PtdS	Phosphatide	792.07Da	TREM2 Ig domain hydrophobic pocket	Polar lipids are considered to be endogenous ligands of TREM2	Preclinical primary myeloid cells, mice	Neurodegeneration, peripheral inflammatory injury	Primary mouse microglia, 5XFAD, mouse demyelination model	(58)
	AL002	Humanized IgG1 mAb	/	Human TREM2 ectodomain; AL002c maps to aa112-174	Agonizes TREM2; decreases CSF sTREM2 and increases osteopontin, indicating sustained CNS target engagement	Phase II completed; negative INVOKE-2; extension stopped (NCT04592874)	Early Alzheimer’s disease	381 participants; IV every 4 weeks for 48–96 weeks; no CDR-SB benefit; frequent ARIA-like MRI changes	(168)
	4D9	Antibody	/	PPP2R4 and TREM2 stem areas	Residue Asp137-Gly145 binds to the stem region of TREM2 and reduces its proteolytic cleavage	Preclinical, ATV:4D9 BBB-penetrant variant under preclinical development	AD, multiple sclerosis	5XFAD, cuprizone demyelination mice, TfR knock-in humanized mice	(171)
	4B2A3	Antibody	/	TREM2	Unknown	*In vitro* cell tool only	No clinical indication	HEK293-TREM2 overexpression cell line, primary mouse microglia	(171)
	scFv-2	Antibody	/	TREM2 Ig domain	By binding to the Ig domain	*In vitro* cell tool only	No clinical indication	HEK293 human TREM2 stable cell line	(21)
	scFv-4	Antibody	/	TREM2 Ig domain		*In vitro* cell tool only	No clinical indication	HEK293 human TREM2 stable cell line	(21)
	PY314	Antibody	/	TREM2 extracellular ectodomain	Unknown	Clinical Phase I (discontinued NCT04691375)	Lung adenocarcinoma, colorectal cancer, ovarian cancer, breast cancer, renal cell carcinoma, advanced refractory solid tumors	MC38 colon carcinoma, EMT6 breast cancer, RENCA renal cell carcinoma mouse syngeneic tumor models	(184)
Direct antagonists	OIA (TREM2-IN-1)	Pt(IV) complex (oxaliplatin-artesunate)	1164.12 Da	Macrophage TREM2; binding site unresolved	TREM2 inhibition plus DNA platination; reduces CD206+/CX3CR1+ macrophages and increases DC, cytotoxic T and NK cells	Preclinical research compound	Colorectal cancer/solid tumors (preclinical)	Human colon cancer cells and macrophages; MC38 tumor-bearing mice	(175)
	EOS-215	Fully human mAb	/	Ligand-binding surface; epitope undisclosed	Competes with PS/LDL; blocks multimerization, signaling and efferocytosis; reprograms TREM2+ macrophages	Phase I/Ib terminated for sponsor strategy (April-July 2025; NCT06877533)	Advanced solid tumors; monotherapy or pembrolizumab combination	4T1 and CT26 models; 3 trial participants; no results posted	AACR 2025; NCT06877533
Indirect modulators	PLX3397	Free base	395.41Da	CSF1R (microglia)	By inhibiting the activity of CSF1R in microglia	Preclinical, research compound	Preclinical neurodegeneration research tool	5XFAD AD mice, TREM2 KO crossbred 5XFAD mice, traumatic brain injury mice	(181)
	PLX5622	Free base	417.81Da	CSF1R (microglia)	Inhibits CSF1R-dependent microglial survival, causing transient depletion and repopulation and indirectly reducing the cellular reservoir expressing TREM2	Preclinical, research compound	PD, AD preclinical model intervention	α-Synuclein PFF-injected PD mice, 5XFAD mice	(181)
	ADAM10/17-γ-secretase processing	Protease- pathway modulation	/	TREM2 shedding and intramembrane cleavage	Enhances shedding and/or cleavage to lower membrane TREM2 and alter sTREM2; nonselective protease effects are a major limitation	Conceptual/preclinical; no selective agent	Excess or maladaptive TREM2 signaling (theoretical)	Not specified	(177)
	Exogenous sTREM2/processing-balance modulation	Soluble protein/processing modulation	/	Membrane TREM2-sTREM2 equilibrium	Modulates survival, proliferation and inflammatory genes; exogenous sTREM2 may reduce amyloid pathology, but its signaling role remains unresolved	Preclinical; clinical biomarker evidence	AD; ALS (biomarker context)	AD models; specific model not stated	(185)
	ASO-mediated expression modulation	Antisense oligonucleotide	/	TREM2 transcript/MS4A-related regulators	Modulates deleterious transcripts or upstream control of TREM2 expression	Conceptual/preclinical; CNS delivery unresolved	TREM2 dysfunction, including disease-associated variants	Not specified	Not available

### 5.2 TREM2 inhibition strategies and indirect modulation

To date, clinically validated and highly selective TREM2 inhibitors have not been established, reflecting both the structural complexity of the receptor and the essential physiological functions of TREM2 in maintaining microglial homeostasis. Unlike conventional inflammatory targets, complete inhibition of TREM2 signaling may produce undesirable consequences by impairing microglial survival, lipid metabolism, lysosomal function, and tissue surveillance. Therefore, therapeutic strategies aimed at reducing TREM2 activity should not be considered as a general approach for CNS disorders, but rather as a potential precision intervention for pathological conditions in which persistent or excessive TREM2 activation contributes to maladaptive microglial remodeling.

Recent studies have provided initial proof-of-concept evidence for direct pharmacological inhibition of TREM2. A platinum-based small-molecule compound, termed TREM2-IN-1 (OIA), was reported to suppress TREM2-mediated signaling and remodel immunosuppressive myeloid programs within the tumor microenvironment, representing one of the first attempts to directly target TREM2 activity (175). However, its molecular binding mechanism, selectivity toward TREM2 over other myeloid receptors, and applicability beyond oncology remain insufficiently characterized. Similarly, EOS-215, an antagonistic anti-TREM2 monoclonal antibody developed by iTeos Therapeutics, has emerged as another potential TREM2-blocking strategy. Preliminary preclinical data presented at AACR suggested that EOS-215 could interfere with TREM2 ligand engagement and downstream macrophage activation programs (176). Nevertheless, peer-reviewed evidence remains limited, and whether these approaches can achieve beneficial modulation of microglial responses in neurodegenerative diseases remains unknown.

Given these limitations, current approaches to attenuate TREM2 signaling remain primarily indirect and focus on regulating receptor abundance, maturation, trafficking, or downstream signaling rather than directly blocking receptor activity. One potential strategy involves targeting TREM2 post-translational processing. TREM2 undergoes regulated ectodomain shedding mediated by ADAM10/17, followed by γ-secretase-dependent intramembrane cleavage, processes that determine the relative abundance of membrane-bound TREM2 and sTREM2. Enhancing receptor shedding or promoting degradation of membrane-associated TREM2 could theoretically reduce surface receptor availability and attenuate downstream signaling (177). However, because ADAM10/17 and γ-secretase regulate numerous additional substrates involved in neuronal function and immune regulation, manipulation of this pathway requires careful evaluation of specificity and potential adverse effects.

Another indirect regulatory strategy involves modulation of the TREM2-DAP12 signaling complex. DAP12 serves as the essential adaptor mediating ITAM-dependent signal transduction downstream of TREM2 and contributes to receptor stability, membrane localization, and downstream SYK activation. Disrupting the TREM2-DAP12 interaction may therefore represent a mechanism to selectively reduce receptor signaling output. However, DAP12 is shared by multiple myeloid immunoreceptors, including other receptors involved in microglial surveillance and innate immune regulation. Consequently, targeting DAP12 alone may compromise broader immune functions and lacks sufficient specificity for clinical application.

Importantly, the potential value of TREM2 inhibition may depend strongly on disease context. In disorders dominated by inadequate clearance capacity, such as early amyloid pathology or acute tissue injury, suppressing TREM2 activity may be detrimental by limiting debris removal and metabolic adaptation. In contrast, in pathological settings characterized by persistent microglial activation, chronic inflammatory remodeling, or maladaptive DAM-like states, partial attenuation of excessive TREM2 signaling may theoretically restore immune balance. For example, prolonged TREM2 activation during certain tau-driven neurodegenerative conditions may contribute to sustained inflammatory remodeling rather than effective tissue repair, suggesting that selective modulation rather than complete inhibition may represent a more rational therapeutic strategy.

Cumulatively, although direct TREM2 antagonists are beginning to emerge, their pharmacological maturity and applicability to CNS diseases remain limited. Current inhibition strategies are largely indirect and must be interpreted within the broader framework of TREM2-dependent microglial adaptation. Future approaches should avoid indiscriminate suppression of TREM2 activity and instead aim to selectively restrain maladaptive signaling while preserving essential homeostatic functions. Achieving this balance will require integration of disease-stage biomarkers, microglial state profiling, and mechanistic understanding of when TREM2 signaling transitions from adaptive regulation to pathological activation.

### 5.3 Microglial replacement and gene-based repair strategies

For disease-associated TREM2 variants, particularly loss-of-function mutations such as R47H, therapeutic strategies are increasingly shifting from modulation of downstream pathways toward restoration of endogenous TREM2 function. Unlike pharmacological approaches that primarily enhance residual receptor activity, cell-based replacement and gene-based repair strategies aim to re-establish the intrinsic neuroimmune–metabolic programs governed by TREM2. These approaches are conceptually based on the observation that TREM2 deficiency disrupts microglial lipid metabolism, phagocytic competence, mitochondrial fitness, and disease-associated transcriptional programs across multiple CNS disorders. Consequently, restoring functional TREM2 may simultaneously normalize immune surveillance, metabolic adaptation, and tissue repair.

**Microglial replacement and population remodeling.**

Among cell-based approaches, iPSC-derived microglia have emerged as valuable platforms for modeling TREM2 deficiency and evaluating functional rescue strategies (178). In preclinical studies, transplantation of genetically intact microglia-like cells partially restores phagocytic capacity, improves lipid handling, attenuates neuroinflammatory responses, and reduces pathological protein accumulation. Functional benefits have been demonstrated in models of both AD and ALS, where engrafted microglia contribute to improved neuronal integrity and behavioral outcomes (179). These findings provide proof-of-concept that replenishing functional microglia can partially re-establish TREM2-dependent neuroimmune homeostasis.

Nevertheless, current replacement strategies remain constrained by several biological challenges. Transplanted microglia do not fully recapitulate the developmental origin, regional identity, or long-term niche adaptation of resident yolk sac-derived microglia. Questions regarding engraftment efficiency, regional heterogeneity, epigenetic maturation, and durable functional integration therefore remain unresolved, limiting the translational readiness of this approach (180).

A complementary strategy involves transient depletion and repopulation of endogenous microglia, most commonly achieved using CSF1R inhibitors such as PLX5622 (181). Rather than directly targeting TREM2, this approach resets the microglial compartment by eliminating dysfunctional populations and permitting repopulation from surviving or progenitor-like microglia (182, 183). Such remodeling may be particularly relevant in chronic neurodegenerative disorders, where persistent DAM progressively lose homeostatic functions and adopt maladaptive inflammatory phenotypes. Although repopulated microglia frequently reacquire homeostatic transcriptional signatures, it remains unclear whether they fully restore TREM2-dependent immunometabolic functions or provide durable therapeutic benefit. Moreover, because microglia are indispensable for synaptic remodeling, immune surveillance, and metabolic homeostasis, global depletion is unlikely to represent a TREM2-specific therapeutic strategy and should instead be regarded as an experimental platform for microglial reprogramming.

**Gene-based repair and expression restoration**.

Direct correction of TREM2 dysfunction has emerged as another promising therapeutic direction. Genome-editing technologies, including CRISPR/Cas systems, offer the possibility of correcting pathogenic TREM2 variants and restoring endogenous receptor signaling, whereas antisense oligonucleotides (ASOs) provide a complementary strategy for modulating TREM2 expression or targeting upstream regulatory pathways, including members of the MS4A locus that influence TREM2 expression. Compared with pharmacological agonists, these genetic approaches have the potential to restore physiological TREM2 signaling rather than simply augment downstream pathway activity. However, substantial technical challenges continue to limit clinical translation. Efficient delivery to resident microglia, maintenance of long-term expression stability, avoidance of off-target genomic effects, and preservation of cell-type specificity remain major obstacles. Furthermore, the therapeutic efficacy of gene-based interventions is likely to depend on disease stage, microglial activation state, and blood–brain barrier integrity, all of which influence target accessibility and biological responsiveness.

**Translational considerations**.

A common challenge shared by both cell-based and gene-based strategies is the requirement for efficient and selective delivery to microglia within the CNS. Although viral vectors, lipid nanoparticles, and engineered extracellular vesicles have substantially improved CNS delivery in experimental models, their clinical applicability remains limited by heterogeneous biodistribution, variable microglial transduction efficiency, immunogenicity, and disease-dependent alterations of the CNS microenvironment. These limitations suggest that successful therapeutic implementation will require not only advances in delivery technologies but also improved patient stratification and disease-stage-specific intervention. Together, microglial replacement and gene-based repair represent next-generation therapeutic strategies aimed at restoring the endogenous neuroimmune–metabolic functions of TREM2 rather than merely modulating downstream inflammatory pathways. While current evidence provides compelling proof-of-principle for functional rescue in preclinical models, substantial challenges remain before these approaches can be translated into clinical practice. Future therapeutic development will likely rely on integrated strategies combining precision gene editing, engineered microglial replacement, targeted delivery systems, and disease-stage-specific intervention to achieve durable restoration of TREM2-dependent microglial homeostasis.

### 5.4 TREM2 and sTREM2 in the regulation of neuroinflammatory networks

TREM2 functions as a central regulator of neuroimmune homeostasis by coordinating inflammatory activation, phagocytic clearance, and tissue remodeling rather than acting as a simple pro- or anti-inflammatory receptor. Through DAP12-dependent signaling, TREM2 integrates multiple extracellular danger signals with intracellular immune programs, thereby shaping microglial activation states according to disease stage and local microenvironment. Increasing evidence indicates that TREM2 signaling interacts extensively with TLR pathways, complement cascades, and stress-responsive MAPK signaling, positioning TREM2 as a key coordinator of inflammatory adaptation rather than a binary regulator of inflammation. In particular, TREM2 restrains excessive TLR-mediated inflammatory responses while simultaneously promoting the clearance of apoptotic neurons, myelin debris, lipid-rich particles, and protein aggregates, thereby facilitating resolution of neuroinflammation and restoration of tissue homeostasis.

Among these interacting pathways, the complement system represents one of the most important downstream interfaces linking TREM2 to synaptic remodeling and chronic neuroinflammation. Experimental studies suggest that TREM2 influences complement-mediated microglial functions through the C1q-C3-C3aR signaling axis (186), thereby regulating synaptic pruning, immune cell recruitment, and inflammatory amplification. Because complement activation and TREM2 signaling are both dynamically regulated during disease progression, their functional relationship appears highly context dependent and varies across pathological stages and CNS regions. Rather than functioning in parallel, current evidence supports the concept that TREM2 and complement signaling constitute an integrated neuroimmune regulatory network controlling both inflammatory resolution and tissue remodeling.

Beyond membrane-associated signaling, TREM2 activity is further regulated through ectodomain shedding, which generates sTREM2 following ADAM10/17-mediated proteolytic cleavage. Increasing clinical evidence consistently demonstrates elevated cerebrospinal fluid sTREM2 concentrations in AD, ALS, and several other neurodegenerative disorders, where sTREM2 levels correlate with disease progression, microglial activation, and biomarkers of neurodegeneration. These observations have established sTREM2 as a promising indicator of disease-associated microglial states rather than simply a by-product of receptor cleavage.

Mechanistically, accumulating evidence suggests that sTREM2 possesses intrinsic biological activity. Depending on the pathological context, sTREM2 has been reported to promote microglial survival, proliferation, phagocytic competence, and inflammatory remodeling while influencing downstream transcriptional programs associated with immune adaptation. Experimental administration of recombinant sTREM2 reduces amyloid deposition, enhances microglial clustering around plaques, and improves cognitive performance in several AD models, supporting its active regulatory role within the neuroimmune microenvironment. Nevertheless, whether sTREM2 primarily functions as an autonomous signaling ligand, a competitive decoy receptor, or a context-dependent immunomodulator remains incompletely resolved and is likely influenced by disease stage, ligand availability, and the surrounding inflammatory milieu.

Importantly, membrane-bound TREM2 and sTREM2 should not be viewed as independent entities but rather as components of a dynamic regulatory equilibrium that fine-tunes neuroimmune homeostasis. Excessive inhibition of TREM2 ectodomain shedding may preserve membrane receptor signaling while simultaneously limiting potentially beneficial functions mediated by sTREM2, whereas excessive shedding may reduce cell-surface receptor availability despite increasing extracellular sTREM2 concentrations. Consequently, therapeutic manipulation of TREM2 processing requires balancing these complementary signaling modalities rather than selectively maximizing either membrane-bound or soluble TREM2.

Collectively, current therapeutic strategies targeting the TREM2 pathway operate at multiple regulatory levels, including receptor activation, modulation of intracellular signaling cascades, regulation of receptor processing and sTREM2 generation, and restoration of microglial function through gene- and cell-based approaches. Rather than representing independent therapeutic paradigms, these interventions aim to re-establish neuroimmune-metabolic homeostasis. Because the biological consequences of TREM2 modulation vary substantially according to disease stage, pathological substrate, and microglial activation state, universal receptor activation or inhibition is unlikely to provide optimal efficacy across all CNS disorders. Future translational development will therefore require biomarker-guided precision strategies integrating TREM2 genotype, CSF sTREM2 dynamics, disease-specific pathological signatures, and temporal windows of therapeutic intervention to maximize clinical benefit.

## 6 Concluding remarks and future directions

As a microglia-enriched immunoreceptor, TREM2 has emerged as a central regulator of CNS immune homeostasis by integrating environmental sensing, metabolic adaptation, phagocytic competence, and inflammatory remodeling. Through coordinated signaling networks involving DAP12-SYK, PLCγ2, PI3K-AKT-mTOR, MAPK, and JAK-STAT pathways, TREM2 enables microglia to transition between homeostatic surveillance states and disease-associated functional programs. However, accumulating evidence across diverse CNS disorders indicates that TREM2 should not be regarded as an inherently protective or detrimental molecule. Instead, TREM2 functions as a context-dependent regulator of microglial adaptability, whose biological consequences are determined by the pathological substrate, disease stage, signal intensity and duration, cellular environment, and metabolic capacity of responding microglia.

Across neurological disorders, the functional role of TREM2 is shaped by disease-specific pathological demands. In AD, TREM2 primarily regulates microglial responses at the interface of Aβ deposition, ApoE-dependent lipid dysregulation, and synaptic remodeling, where appropriate activation promotes plaque containment and metabolic adaptation but may become maladaptive during tau-dominant progression. In PD, TREM2 is closely linked to α-Syn handling, autophagy-lysosomal competence, and mitochondrial stress adaptation, with its protective effects depending on maintaining microglial capacity for proteostatic clearance. In ALS, TREM2 mainly controls microglial state transitions in response to continuous motor neuron stress and intracellular protein aggregation, whereas in ischemic injury, TREM2 coordinates acute debris clearance, inflammatory resolution, and subsequent tissue repair. These disease-specific functions highlight that the therapeutic relevance of TREM2 depends not simply on the magnitude of receptor activation, but on whether signaling occurs within an adaptive or maladaptive cellular state.

Despite substantial progress, several fundamental questions remain unresolved. First, the molecular mechanisms defining the transition between beneficial and detrimental TREM2 signaling remain poorly understood. The critical thresholds that determine when TREM2 activation supports tissue protection versus when it promotes persistent inflammatory remodeling have not been clearly established. Second, the functional relationship among membrane-bound TREM2, sTREM2, and downstream signaling branches requires further clarification. Although sTREM2 has emerged as an important biomarker, it remains uncertain whether alterations in circulating or CSF sTREM2 primarily represent receptor shedding, changes in microglial abundance, compensatory activation, or an independent immunomodulatory mechanism. Third, the contribution of disease-associated TREM2 variants and their impact on ligand recognition, receptor signaling capacity, and therapeutic responsiveness remains incompletely defined. Finally, the interaction between TREM2-dependent microglial states and other cellular components, including astrocytes, peripheral immune cells, synaptic networks, and vascular elements, requires further mechanistic dissection.

Future studies should therefore move beyond static assessment of TREM2 expression or single inflammatory markers and instead define the dynamic cellular states regulated by TREM2 during disease progression. Integrative approaches combining single-cell multi-omics, spatial transcriptomics, proteomics, metabolomics, and advanced imaging will be essential for resolving the spatial and temporal evolution of TREM2-associated microglial programs. Importantly, these approaches should incorporate human-relevant experimental systems, including patient-derived iPSC microglia, human brain organoids, xenotransplantation models, and longitudinal clinical cohorts, because fundamental differences exist between human and murine microglial biology. In particular, species-specific regulation of DAM states, lipid metabolism, and TREM2-dependent transcriptional programs represents a major barrier to direct translation of findings obtained from experimental models.

The clinical development of TREM2-targeted therapies faces additional challenges. The limited efficacy of recent clinical trials despite strong preclinical rationale indicates that receptor engagement alone is insufficient to restore functional microglial homeostasis. Most current therapeutic strategies have been developed in amyloid-centered models and may not fully capture the complexity of human neurodegenerative diseases, including tau propagation, chronic inflammatory remodeling, aging-associated metabolic dysfunction, and genetic heterogeneity. Furthermore, broad modulation of TREM2-associated pathways may affect essential microglial functions, including immune surveillance, lipid metabolism, and tissue maintenance. Therefore, future therapeutic strategies must carefully consider not only target engagement but also restoration of functional microglial outputs, including clearance capacity, lysosomal activity, metabolic fitness, and inflammatory resolution.

Accordingly, the future of TREM2-based therapy will likely depend on precision modulation rather than generalized activation or inhibition. Effective intervention will require identifying when, where, and in which patients TREM2 signaling remains therapeutically reversible. Biomarker-guided approaches integrating TREM2 genotype, ApoE status, sTREM2 dynamics, pathological protein profiles, neuroimaging features, and immune-metabolic signatures may enable the identification of therapeutic windows during which TREM2 modulation is most beneficial. In addition, combination strategies targeting complementary pathological processes, including protein aggregation, complement activation, lipid dysregulation, mitochondrial dysfunction, and metabolic failure, may provide greater therapeutic benefit than isolated TREM2 manipulation.

In conclusion, TREM2 represents a biologically complex but highly informative therapeutic axis connecting immune surveillance, metabolic adaptation, proteostasis, and tissue remodeling in CNS disorders. The translational potential of TREM2 is unlikely to depend on maximizing receptor activity, but rather on restoring the appropriate microglial response within a specific disease context, temporal window, and cellular environment. Defining the spatiotemporal and cell-state-dependent principles governing TREM2 function will be essential for transforming mechanistic insights into safe and clinically effective interventions for neurological diseases.
